# Glycomic and Glycoproteomic Techniques in Neurodegenerative Disorders and Neurotrauma: Towards Personalized Markers

**DOI:** 10.3390/cells11030581

**Published:** 2022-02-08

**Authors:** Firas Kobeissy, Abir Kobaisi, Wenjing Peng, Chloe Barsa, Mona Goli, Ahmad Sibahi, Samer El Hayek, Samar Abdelhady, Muhammad Ali Haidar, Mirna Sabra, Matej Orešič, Giancarlo Logroscino, Stefania Mondello, Ali H. Eid, Yehia Mechref

**Affiliations:** 1 Department of Biochemistry & Molecular Genetics, Faculty of Medicine, American University of Beirut, Beirut 1107 2020, Lebanon; firasko@gmail.com (F.K.); abeerkobaisi@gmail.com (A.K.); cab20@mail.aub.edu (C.B.); ams105@mail.aub.edu (A.S.); muhammad.a.haidar@gmail.com (M.A.H.); 2 Department of Chemistry and Biochemistry, Texas Tech University, Lubbock, TX 79409, USA; wenjing.peng@ttu.edu (W.P.); mona.goli@ttu.edu (M.G.); 3 Department of Psychiatry, Faculty of Medicine, American University of Beirut, Beirut 1107 2020, Lebanon; samer.elhayek@gmail.com; 4 Faculty of Medicine, Alexandria University, Alexandria 21544, Egypt; samar.abdelhady606@gmail.com; 5 Neuroscience Research Centre, Faculty of Medical Sciences, Lebanese University, Beirut 6573, Lebanon; myrnasabra@hotmail.com; 6 School of Medical Sciences, Örebro University, SE-701 82 Örebro, Sweden; matej.oresic@oru.se; 7 Turku Bioscience Centre, University of Turku and Åbo Akademi University, FI-20520 Turku, Finland; 8 Department of Basic Medical Sciences, Neuroscience and Sense Organs, University of Bari, 70121 Bari, Italy; giancarlo.logroscino@neurol.uniba.it; 9 Department of Biomedical and Dental Sciences and Morphofunctional Imaging, University of Messina, 98125 Messina, Italy; stefania.mondello@unime.it; 10 Department of Basic Medical Sciences, College of Medicine, QU Health, Qatar University, Doha P.O. Box 2713, Qatar; 11 Biomedical and Pharmaceutical Research Unit, QU Health, Qatar University, Doha P.O. Box 2713, Qatar

**Keywords:** glycosylation, post-translational modifications, neurodegenerative diseases, neuropsychiatric disorders, proteomics

## Abstract

The proteome represents all the proteins expressed by a genome, a cell, a tissue, or an organism at any given time under defined physiological or pathological circumstances. Proteomic analysis has provided unparalleled opportunities for the discovery of expression patterns of proteins in a biological system, yielding precise and inclusive data about the system. Advances in the proteomics field opened the door to wider knowledge of the mechanisms underlying various post-translational modifications (PTMs) of proteins, including glycosylation. As of yet, the role of most of these PTMs remains unidentified. In this state-of-the-art review, we present a synopsis of glycosylation processes and the pathophysiological conditions that might ensue secondary to glycosylation shortcomings. The dynamics of protein glycosylation, a crucial mechanism that allows gene and pathway regulation, is described. We also explain how—at a biomolecular level—mutations in glycosylation-related genes may lead to neuropsychiatric manifestations and neurodegenerative disorders. We then analyze the shortcomings of glycoproteomic studies, putting into perspective their downfalls and the different advanced enrichment techniques that emanated to overcome some of these challenges. Furthermore, we summarize studies tackling the association between glycosylation and neuropsychiatric disorders and explore glycoproteomic changes in neurodegenerative diseases, including Alzheimer’s disease, Parkinson’s disease, Huntington disease, multiple sclerosis, and amyotrophic lateral sclerosis. We finally conclude with the role of glycomics in the area of traumatic brain injury (TBI) and provide perspectives on the clinical application of glycoproteomics as potential diagnostic tools and their application in personalized medicine.

## 1. Introduction

### 1.1. Post-Translational Modifications (PTMs)—An Overview

The proteome represents all the proteins expressed by a genome, a cell, a tissue, or an organism at any given time under defined physiological circumstances [1]. Even though proteins result from the well-known two-step process of transcription then translation of specific nucleotide sequences, these macromolecules, which are the building blocks of life, can and will undergo several modifications throughout their life span. Such alterations, known as post-translational modifications (PTMs), affect numerous protein properties/characteristics that modify protein functions, including [2] protein lifespan, solubility, folding, localization, abundance, protein-protein interactions, [3] receptor activation, enzyme function, and assembly [4]. 

Advances in the molecular biology field, mainly in genomics and proteomics, pave the way for understanding the mechanisms underlying various PTMs [5,6]. Presently, the number of PTMs discovered has exceeded 400, and research for the past decade has established the important role that such modifications play in various biological processes such as signal transduction, gene expression regulation, DNA repair, and cell cycle regulation [7]. Table 1 inserted below summarizes the main characteristics of the essential PTMs.

Among these PTMs, glycosylation is a major PTM associated with many important biological processes such as receptor activation and signal transduction, protein folding and degradation, as well as cell adhesion and cell-to-matrix interaction is glycosylation [8,9]. This modification that results from the attachment by the covalent bond of an oligosaccharide chain residue commonly targets serine (Ser), threonine (Thr), asparagine (Asn), and tryptophan (Trp) sites is catalyzed by a glycosyltransferase enzyme, and it may occur in the ER, the Golgi apparatus, or the cytosol [10].

### 1.2. Glycosylation of Proteins

**Figure 1:**
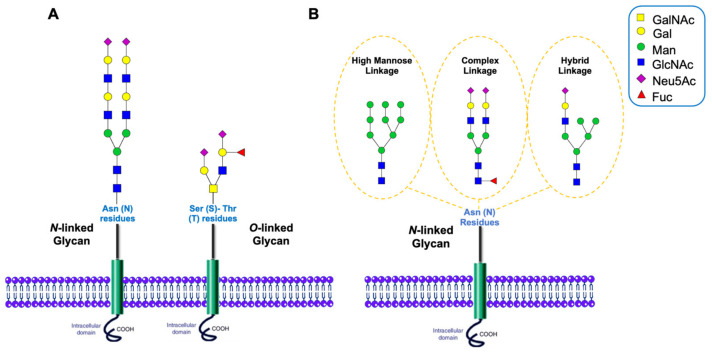
A depiction of the nomenclature, topology, and glycosylation patterns of *N*- and *O*-glycans. (**A**) Linkage of *N*-acetylglucosamine to asparagine amino acid via an *N*-linked bond, followed by linkage of *N*-acetylgalactosamine to serine or threonine amino acids via an *O*-linked bond. The glycoprotein shown is a transmembrane protein. The possible bonds formed between glycan residues are illustrated. (**B**) The three possible types of *N*-linked glycosylation products, depicted through transmembrane proteins. GlcNAc: *N*-acetylglucosamine; Man: mannose; Gal: galactose; NeuNAc/Sia: *N*-acetylneuraminic acid/sialic acid; Fuc: fucose.

As previously mentioned, one of the most common PTMs is glycosylation, as it embodies more than half of the mammalian cell protein modifications [11], with 70% of eukaryotic proteins having undergone at least one glycosylation [12]. This process of attachment of a sugar/carbohydrate moiety to certain sites of organic molecules, such as lipids or proteins, offers greater proteomic diversity when compared to other modifications [13,14]. Table 2, inserted right below, describes the different types of glycosylation. From the literature, it has been established that protein glycosylation mediates a series of important biological functions such as cell communication [15,16], adhesion [14,17,18], trafficking [19,20], and protein stabilization [21]. Research has shown that glycosylation is a tightly regulated process where specific substrate/enzymatic modifications occur in particular organelles [22]. There are two main types of glycosylation, *N*- and *O*-glycosylation, as shown in Figure 1 below. *N*-glycosylation refers to the attachment of glycans to the asparagine with a motif of asparagine-X-serine/threonine, where X denotes any amino acids but proline. In contrast, *O*-glycosylation refers to the attachment of glycans to the serine/threonine without a consensus motif [22,23,24,25,26]. 

### 1.3. Dynamics of Glycosylation

Glycosylation is known to be a template-free process regulated by modifying metabolic enzymes like glycosyltransferases [27,28]. Human glycans mainly contain different combinations of the following monosaccharide units: mannose (Man), glucuronic acid (GlcA), galactose (Gal), glucose (Glc), *L*-fucose (Fuc), *N*-acetylgalactosamine (GalNAc), sialic acid (Sia, Neu5Ac), and *N*-acetylglucosamine (GlcNAc) [29,30]. The different resulting sugar combinations alongside the various possible isomerization patterns that will follow—whether positional or linkage isomerization—introduce and add to the richness and the vast microheterogeneity of glycosylation [31].

Glycobiological and glycoproteomics studies over the years have revealed that the dynamic process of glycosylation determines important events such as cell-cell interaction, cellular metabolism, and extracellular communication [32,33]. Almost all secreted proteins and intracellular proteins are amended by the addition of oligosaccharides, where glycans are covalently bonded to proteins to regulate their final structure and function [33,34]. The reasons behind glycosylation vary, including modulation and structural adaptation, intrinsic and extrinsic recognition, and mimicry of other glycosylated proteins in hosts [35]. Thus, glycans are said to be “turned on and off” to modulate glycoproteins [36]. 

Two cellular components are essential to glycosylation—endoplasmic reticulum (ER) and Golgi apparatus. Glycosylation is initiated in ER by forming the conserved precursor oligosaccharide. *N*-glycan precursors will be further processed in the ER to remove terminal glucoses before being transferred to Golgi. [37]. Therefore, proper ER structure and functioning are both needed and required for proper glycosylation dynamics. The glycans will then be modified by different glycosidases and glycosyltransferases to form complex structures. Many in vitro studies have revealed the impairment of protein glycosylation due to the disruption of Golgi’s structure [38,39]. Mass-spectrometry analysis of GRASP-depleted HeLa cells revealed decreased levels of high-mannose and complex glycans, thus divulging the importance of Golgi structure in maintaining protein glycosylation [40].

### 1.4. Pathophysiological Aspects of Mis-Glycosylated Products

Glycosylation is crucial in allowing genes and pathways to function properly. Any mutation present in glycosylation-related genes may lead to the formation of neurologically impaired individuals. These mutations, specifically the congenital disorders of glycosylation (CDG), have been proven to participate in the occurrence of over 80% of neurological abnormalities [41]. Glycans can present irregularities on either proteins or lipids, leading to various genetic defects. Within a mammalian cell, the glycome is highly complex, even more so than the proteome or the genome [42]. This complexity provides a fine-tuning mechanism for several cellular processes, where different proteins are expressing the same sugar chain and present diverse functional consequences. The outcome of glycosylation is mostly context-dependent [43]; several factors influence the formation of the final glycosylation product. These include the supply of the activated sugars, the identity of the proteins or lipids attached, and the enzymes involved in the biosynthesis [44]. Glycosylated proteins can be connected to several different glycan types, making each form a unique one employed in specific pathways [45]. Consequently, any hindrance preventing their formation or delivery can affect the related glycosylation pathways. 

Proper glycosylation necessitates the correct functioning of the Golgi system. Flaws in the trafficking of proteins and their composition along with unstable Golgi homeostasis may directly impact glycosylation. Trafficking defects may be due to the mislocalization of several glycosyltransferases and nucleotide-sugar transporters, impacting single or multiple glycosylation pathways. These defects mainly transpire in cytoplasmic proteins transiently associated with the Golgi system, hence affecting the guidance of vesicles holding glycosylation machinery to their location [46]. 

Other glycosylation defects may be seen during aging, which is related to the onset of several diseases [47]. Glycosylation can endure age-related modifications, subsequently increasing molecular heterogeneity and impaired protein function, such as in the case of age-related pathologies including sarcopenia and cataracts [48].

Many diseases can be correlated to defective glycosylation. Liver diseases such as hepatitis C virus (HCV) or hepatitis B virus (HBV) are thought to be caused by hyper fucosylation, increasing the branching, and bisecting the *N*-acetylglucosamine present in glycans [49]. These chronic infections can then lead to hepatocellular carcinoma and cirrhosis in humans. The alterations of different glycans have been observed in a variety of diseases such as liver disease [50,51], diabetes [52,53], and gestational diabetes mellitus (GDM) [54]. 

The most commonly widespread glycosylation disorder is manifested through phosphomannomutase 2 (PMM2) mutation, which transforms mannose-6-phosphate to mannose-1-phosphate. This defect, in turn, reduces the products used for *N*-glycosylation, leaving many proteins unstable due to the partly employed *N*-glycosylation sites [44]. The PMM2 mutation can lead to several neurological defects in children characterized as *N*-linked disorders such as hypotonia, intellectual disabilities, stroke-like episodes, and seizures [55]. 

Another mutation occurring at the NGLY gene that encodes cytoplasmic enzymes can cause what is known as the “congenital disorder of de-glycosylation”. This specific mutation hampers the normal progression of the endoplasmic reticulum-associated degradation (ERAD) pathway, leading to the hindrance in the degradation of misfolded *N*-glycosylated proteins. Patients with mutated NGLY1 show movement disorders, microcephaly, and developmental delays [56]. Other diseases showing glycosylation defects include asthma, chronic pain, arthritis, and amyotrophic lateral sclerosis (ALS) [57,58].

Glycosylation studies have become significant in uncovering the various aspects of cancer. The role of glycoproteins and glycans has become prominent in cancer studies, where glycosylation is thought to be associated with carcinogeneses such as metastasis, tumor adhesion, and malignant transformation [59]. Recent advances in glycomics have prompted the discovery of unique *N*- and *O*-glycans acting as glycobiomarkers used in cancer diagnosis and therapy [60].

## 2. Glycomics and Glycoproteomics Methodology

### 2.1. Challenges in Glycomics and Glycoproteomics

Although glycoproteomic studies have been attracting more interest in recent decades, the challenge of this cutting-edge analysis remains significant due to the fact that (i) glycoproteins usually present in low abundance relative to common proteins in a biological system; (ii) ionization efficiency of glycans is poor relative to peptides and proteins, and (iii) glycans have no UV or fluorescent absorbance, thus UV detectors and fluorescence detectors are no longer effective in glycomic analysis. Therefore, different technologies and approaches have been developed to overcome these challenges, improving the sensitivity and reliability of glycomic and glycoproteomic characterizations. There are two major strategies for studying protein glycosylation: glycomics and glycoproteomics. Glycomic research analyzes glycans released from biological sources, while glycoproteomic research focuses on the characterization of intact glycoproteins and glycopeptides. Although glycoproteomics provides information on both glycoforms and their occupancies on protein sites, whereas glycomics can only analyze glycans, glycomics has higher sensitivity and separation efficiency with the combination of several derivatization and separation techniques, which benefit the characterization of minor structures and isomers. Therefore, both glycomics and glycoproteomics are important in glycoscience and complementary to each other. 

### 2.2. Enrichment of Glycoproteins

Due to the low abundance of glycoproteins in biological samples such as blood serum and tissues, enrichment is necessary prior to glycoproteomics analysis to enhance the signal intensity of glycopeptides. In addition, the microheterogeneity of glycosylation sites and glycan structures, as well as the low ionization efficiency relative to unmodified proteins, also demand an enrichment procedure. During the last decades, many enrichment strategies have been developed and applied in glycoproteomic studies.

#### 2.2.1. Lectin Enrichment

Lectins are specialized proteins that can specifically bind to carbohydrates, recognizing sugar groups of other molecules [61]. They have been widely used to enrich certain types of glycoproteins from biological systems due to their selectivity against different glycan moieties. The most frequently employed lectins are concanavalin A (ConA) and wheat germ agglutinin (WGA) [61]. 

In addition to ConA and WGA, other lectins have been utilized for glycoprotein enrichment, such as lentil lectin (LcH, specific to fucosylated core), elderberry lectin (SNA, specific to sialic acid), Ricinus communis agglutinin (RCA, specific to galactose), and peanut agglutinin (PNA, galactose). Jacalin (AIL) lectin is commonly utilized in *O*-glycopeptide enrichment due to its specificity to GalNAc core structures [61,62]. Lectin materials have been immobilized to different bases such as monolithic resin, magnetic beads, and microarray to achieve a better enrichment and purification efficiency of glycopeptides. However, lectin enrichment suffered from the fact that only a particular type of glycopeptides could be enriched by one lectin material due to the different selectivity of different lectins. To overcome this drawback, multiple lectin combination strategies have been used to enrich a broad type of glycopeptides [63,64].

#### 2.2.2. HILIC Enrichment

Hydrophilic interaction chromatography (HILIC) is widely utilized to enrich glycopeptide due to the fact that glycan is more hydrophilic than a peptide. Glycopeptides have stronger interactions with a highly polar stationary phase such as amine-, hydroxyl-, amide- and zwitterionic particles, allowing the purification of glycopeptides through a binary gradient [65]. Recently, several different types of HILIC materials have been developed to reduce the cost and enhance the enrichment efficiency, including cotton [66,67,68], metal-organic frameworks (MOFs) [69,70], polymers [71,72], and magnetic materials [73,74]. Alternatively, electrostatic repulsion-hydrophilic interaction chromatography (ERLIC) has been employed to enrich glycopeptide. [75]. Recently, Mechref and colleagues investigated the enrichment efficiency of HILIC and ERLIC on breast cancer cell lines and reported complementary results of these methods [76]. With the efficient, non-specific, easy-to-handle, and MS-compatible features, HILIC enrichment has been considered as one of the most widely used approaches in glycopeptide enrichment.

#### 2.2.3. Hydrazide Chemistry Enrichment

Hydrazide chemistry was introduced to extract glycoproteins based on a covalent immobilization on solid hydrazide support [77]. In this method, cis-diol groups of glycans are initially oxidized to form aldehydes and covalently bind to support. After enzymatic digestion (mostly tryptic digestion), non-glycosylated peptides were washed off and glycopeptides were released by PNGase F. This technique has been applied commonly to investigate glycoproteins in many diseases such as liver cancer [78], lung cancer [79], and breast cancer [80]. However, although it has a high specificity of glycoprotein enrichment, the irreversible covalent bond between glycans and supports makes it impossible to acquire complete glycan structural information from an intact glycopeptide, thus hindering the application of this method on intact glycopeptide identification.

#### 2.2.4. Click Chemistry Enrichment

Similar to hydrazide chemistry, click chemistry introduces a covalent bond on glycans of glycoproteins. In this technique, azido groups are incorporated into glycan structures through metabolic labeling that provides an orthogonal enrichment method by click chemistry and subsequently biotin and avidin binding to enrich glycoproteins containing the designated azido labels [78]. It was first achieved by a copper-catalyzed cycloaddition reaction [81] and then improved to be easy-to-use copper-free click chemistry with high labeling efficiency and enrichment rate [82]. To date, the most common reagents for this method are per-acetylation unnatural sugar analogs such as AC_4_ManNAz and AC_4_GlcNAz. However, this enrichment method can only apply to living organisms, such as cell cultures or animals, and the application in clinical samples (blood serum or tissue) is limited.

#### 2.2.5. Boronic Acid Enrichment

Boronic acid is an alternative approach for glycopeptide enrichment. Boronic acid can form reversible cyclic boronate esters with cis-diol groups of glycans, attracting much interest in glycoproteomics. This reversibility of boronic acid to bind glycopeptide prompted the functionalization of boronic acid to other materials that have a large surface area, including magnetic carbon nanotubes [83], Fe_3_O_4_ nanoparticles [84], and metal-organic framework [85]. Recently, Wu and coworkers introduced benzoboroxole dendrimer functionalized beads, which could markedly increase glycopeptide coverage than the conventional boronic acid method [86]. Despite the benefit in glycopeptide enrichment brought by this material, the multiple synthetic steps and lack of commercialization prevent the broad application of this method.

### 2.3. Technologies in Glycomics and Glycoproteomics

In recent decades, techniques have been developed and improved for better glycomic and glycoproteomic separation and identification. Conventional UV or fluorescence-based detection methods are no longer efficient in glycomics analysis due to the low absorption of glycans. Although fluorescent tags can be added to obtain visibility, the lack of enough standards and structural information of UV/fluorescent detectors demands a better identification technique. Thus, MS has become the most common technique in glycomics and glycoproteomics research because of its high sensitivity and adequate structural information [87,88,89,90]. However, the fact that glycans have low ionization efficiency and compete with the protons during the ionization process hinders glycomics and glycoproteomics analysis using MS alone. Therefore, different labeling and separation techniques are coupled with MS to acquire a better characterization of glycoforms. 

#### 2.3.1. Lectin Microarray in Glycomics and Glycoproteomics

Lectin, specific to one or more certain monosaccharides moieties, has been used in glycomics and glycoproteomics studies due to its high selectivity, fast analytical speed, and easy-to-use protocols. Since different lectins have different specificities, a series of lectins can be immobilized to a solid support to profile carbohydrate expression patterns in biological samples [91], known as lectin microarray. This technology allows the analysis of glycan profiles with a simple sample preparation procedure, making it useful for the rapid bulk clinical sample analyses. 

A lectin microarray consisting of 45 lectins was recently applied to profile glycan patterns from Influenza A virus hemagglutinin. Besides glycan moieties, different linkages such as α2, 3- and α2, 6-sialylation as well as α1, 3 galactosylation could be distinguished by specific lectins [92], extending its application to isomeric glycomics studies. However, the lectin array can only recognize monosaccharides’ moieties. The lack of detailed structural information for individual glycoforms precludes the comprehensive characterization of glycans and glycopeptides. 

#### 2.3.2. MS-based Glycomics and Glycoproteomics

*MALDI-MS*. Matrix-assisted laser desorption/ionization (MALDI)-mass spectrometry (MS) has been used as a fast analysis approach of glycoform analysis. MALDI-MS is one of the most widely used MS techniques enabling rapid analysis with simple sample preparation steps as well as sufficient structural information. Numerous investigations have been performed to improve MALDI matrix, among which 2,5-dihydroxybenzoic acid (DHB) is considered the most widely used matrix for glycans and glycopeptides [93]. In addition, 1,1,3,3-tetramethylguanidinium (TMG) salts of p-coumaric acid (CA) (G_3_CA) and 3-Aminoquinoline/p-coumaric acid (3-AQ/CA) have also been reported to be efficient matrices in glycomics and glycoproteomics studies [94]. Recently, a nanomaterial has been reported to be a co-matrix in permethylated glycomics analysis, which significantly increased signal intensity and induced a controllable in-source fragmentation [89]. However, MALDI usually introduces unexpected fragments of labile sialic acids [95]. Therefore, derivatization methods such as methylamidation/esterification [96] and permethylation [89] are utilized to stabilize the sialic acid. Besides, these derivatization methods allowed the differentiation of sialic acid linkage isomers through introducing mass shift [96] or different MS^2^ patterns [97]. 

The rapid analytical speed and controlled laser beam permit in situ glycomic analysis of tissue sections using MALDI-imaging. The glycan expression changes could be distinct in different areas of tissue sections [98,99]. The derivatization of dimethylamidation and subsequent amidation also allows the imaging of sialylated linkage isomers on formalin-fixed paraffin-embedded (FFPE) tissues [100]. This technique has revealed the glycan attributes in their native environment. Despite the clear advantages, the unseparated sample resulted in relatively low signal intensity and a more complicated spectrum, which is not suitable for the comprehensive characterization of glycans and glycopeptides. 

*LC-MS*. Liquid chromatography-tandem mass spectrometry (LC-MS/MS) is the most widely used technique for glycomics and glycoproteomics. The separation prior to MS analysis eliminates the ionization competition and removes impurities such as salts in complex biological samples, facilitating the identification and quantitation of glycans and glycopeptides. Reducing end derivatization is commonly utilized for glycomics analysis to improve the ionization efficiency of native glycans. Multiple separation materials and techniques have been developed to better separate and identify glycans and glycopeptides, including reverse phase, hydrophilic interaction chromatography (HILIC), porous graphitic carbon (PGC), and ion exchange. 

The common separation approach for glycoproteomics is reversed-phase LC-MS/MS. Analytes are resolved by their hydrophobic interactions with the stationary particles, which is ideal for the separation of peptides and glycopeptides. C18 column, as the most widely used reversed-phase material, is the main tool for glycopeptides separation. In addition to C18, C4, or C8, which are commonly utilized for top-down glycoproteomic analysis, many studies have reported label-free glycoproteomics [76,78]. However, different labeling techniques have been applied to enhance the separation, ionization, and quantitation of glycopeptides, including methylamidation ((7-Azabenzotriazol-1-yloxy)tripyrrolidinophosphonium hexafluorophosphate (PyAOP)) [101], esterification and amidation [102], and permethylation [103]. Besides, the isobaric tags, such as isobaric tags for relative and absolute quantitation (iTRAQ) and tandem mass tag (TMT) that have been widely used for proteomics, are also efficient in glycopeptides analysis [77,104]. Together with different MS dissociation techniques such as collision-induced dissociation (CID), higher-energy collision dissociation (HCD), electron-transfer dissociation (ETD), electron transfer/higher-energy collision dissociation (EThcD), and ultraviolet photodissociation (UVPD), these developed techniques prompt the comprehensive glycoprotein characterization.

Other than glycoproteomics, C18-LC-MS/MS can also be used for glycomics. However, permethylation is necessary to allow the separation of glycans on the C18 column due to the hydrophilicity of native glycans. Mechref and coworkers have reported the high-temperature separation of permethylated glycans using nanoC18-LC-MS/MS, and partial isomeric separation was also observed [105]. The permethylation coupled to C18 separation permitted a high sensitivity in glycomics analysis with the least quantitative bias compared to other widely used labeling techniques [104]. However, permethylation conditions prevent the use of different tags due to the poor compatibility of permethylation to most of the commercialized reducing end labeling reagents.

HILIC-LC-MS is another efficient and common technique utilized for native and reducing end-labeled glycans. In this technique, hydrophilic interactions, including hydrogen bonding, ionic interactions, and dipole-dipole interactions [106] between analytes and stationary phase, are the driving forces of separation. [107]. Due to the fact that native glycans cannot be efficiently ionized and detected by UV and fluorescence, reducing end derivatization methods are usually used to enhance the ionization and detection of glycans. The developed and widely used derivatization reagents include 2-aminobenzamide (2-AB)/2-aminobenzoic acid (2-AA) [108], 2-amino-pyridine (2-AP), and aniline [109]. Having fluorescent groups, glycans labeled with these reagents can be quantified by the combination of fluorescence detector and MS. In addition, recently, TMT and RapiFluor have been proved to be efficient in LC-MS-based glycomics analysis [104]. Other than derivatization, different HILIC materials are also introduced to improve the separation of glycans. The common HILIC materials for glycomics include zwitterionic (ZIC^®^)-HILIC columns [108,110], amide/amine columns [111,112], and hydroxyl group HILIC columns [113]. Besides separating compositional glycans, HILIC also permits the separation of glycan isomers [110], including positional isomers and linkage isomers [114,115]. 

Besides HILIC, porous graphitic carbon (PGC) is the most widely used material for isomeric separation of both native/reducing end-labeled glycans and permethylated glycans. There are two major forces, dispersive interactions (reverse-phase type) and polar retention effect, that prompt the separation of glycan isomers [116]. Native and reducing end-labeled isomeric *N*- and *O*-glycan separation on PGC has been demonstrated in recent decades [117,118,119,120]. An alternative strategy of PGC-LC-MS is the separation of permethylated glycan isomers. Recently, Mechref and coworkers [104,121] introduced an improved PGC-LC-MS approach at a high temperature (75 °C) to achieve a decent separation of permethylated glycan isomers. This approach was also applied to complex biological samples to investigate the *N*-glycan isomeric changes in diseases [61,122], and separate permethylated free oligosaccharide isomers [123].

Ion exchange chromatography is also used for glycan profiling [124]. Recently, a high-pH anion-exchange chromatography (HPAEC)-MS was used to acquire structural information of glycans [125]. However, the compatibility of HPAEC with MS is a challenge due to the high concentration of salts used in HPAEC that would significantly inhibit the ionization efficiency of analytes. Nevertheless, this issue can be addressed by using an online suppressor that replaces Na^+^ with H^+^. The most common use of ion-exchange chromatography is the fractionation of glycan samples in 2D-LC. Glycans could be separated in the anion exchange column by the size and number of sialic acids [126] and then further separated by another column [127]. Although the major application of ion-exchange chromatography is not the isomeric separation of glycans, it has the ability to resolve several glycan isomers [128,129]. 

*CE-MS*. Capillary electrophoresis (CE)-MS is a powerful tool to study glycans and glycopeptides with a high resolution and a short analytical time. The separation of analytes is based on their sizes, shapes, and charges. The high electric field of CE allows the analysis with an ultrahigh-resolution and a low number of samples. These features make CE-MS capable of analyzing the microheterogeneity of both glycopeptides and intact glycoproteins [130,131,132,133,134]. 

When CE-MS is utilized for glycomics analysis, glycans are usually derivatized prior to CE. The common derivatization reagents that enhance the separation and ionization include 9-aminopyrene-1,3,6-trisulfonic acid (APTS) [135,136], TMT [137] and 2-AA [138,139]. Despite other tags developed for CE-MS, APTS is still the most common reagent for glycomics studies [140]. As a type of CE, a DNA sequencer has also been utilized for glycomics analysis [135,141]. Moreover, CE has proved its ability for isomeric separation of glycans [142,143]. However, the compatibility of electrolytes in CE with MS and the low flow rate hindered most CE methods from being applied in CE-MS. However, improved MS-compatible electrolytes and a sheath flow are used to fix these issues. 

*IM-MS*. Ion mobility (IM), as a gas phase separation technique, provides a secondary separation dimension beyond condensed-phase separation. Ionized analyte molecules are separated in an electric field of a drift cell, thus being resolved by their masses, charges, sizes, and shapes when colliding with buffer gas molecules [142,144]. The drift time of different analyte ions can be converted to rotationally averaged collision cross-section (CCS), thus being independent of instrumental settings and only related to the feature of ions. Besides identifying glycopeptides [145], partial isomeric separation of glycans has been achieved using IM-MS [146,147]. Although IM-MS has attracted much interest in recent years, relatively low resolution hampered its application to characterize glycans and glycopeptides comprehensively. However, its unique separation mechanism could be an additional tool combined with other separation techniques.

#### 2.3.3. Dissociation and Acquisition Techniques Facilitate MS-based Glycomic and Glycoproteomic Identification and Quantitation

One advantage of MS-based glycoproteomics and glycomics is the adequate structural information acquired by various dissociation techniques. The most common dissociation methods are collision-induced dissociation (CID) and higher-energy collisional dissociation (HCD), which have been applied in glycomics [148,149,150,151,152,153] and glycoproteomics [154,155,156] for years. However, as the low-mass limitation (1/3 cutoff) of CID, glycan oxonium ions cannot be detected when analyzing large precursor ions. Compared to CID, HCD overcomes this issue and offers higher resolution [157]. Moreover, the oxonium ions generated in HCD can be employed to trigger other dissociation techniques such as electron transfer dissociation (ETD) in glycoproteomic analysis to increase the dynamic range and duty cycles [158,159]. In addition to CID/HCD, electron transfer dissociation (ETD) and electron capture dissociation (ECD) are significant in glycoproteomic studies since they majorly fragmentate peptide backbone while keeping glycan structures intact [160].

Although the aforementioned techniques are able to provide structural information for the identification of glycopeptides and glycans, they are not efficient in generating cross-ring fragmentation that is necessary for identifying glycan linkage isomers. Thus, there is a great challenge to acquire enough information for isomeric identification of glycan and glycopeptide isomers. To overcome this issue, multiple novel dissociation methods were developed and investigated in this decade, including infrared multiphoton dissociation (IRMPD) [161,162,163], ultraviolet photodissociation (UVPD) [164,165,166], and charge transfer dissociation (CTD) [167,168]. These methods can provide more cross-ring fragmentation, thus facilitating the identification of glycan and glycopeptide isomers. However, the novel dissociation methods were mainly demonstrated on standards or model glycoproteins. It is necessary to further investigate these techniques using complex biological and biomedical samples in future studies.

Another efficient way to improve the identification of glycans and glycopeptides is the combination of different dissociation techniques such as EThcD [169,170], activated-electron photodetachment (a-EPD) [171], and CID/UVPD [172]. In addition to tandem MS, MS^n^ techniques are also employed to improve the identification of glycans [173,174,175] and glycopeptides [176,177], where n usually ranges from 3 to 5. However, the relatively large amount of sample needed for MS^n^ hinders its application to address biological issues where sample amounts are usually limited.

With the aforementioned dissociation techniques, several acquisition strategies can be used to improve the quantitation of glycans and glycopeptides. Multiple reaction monitoring (MRM) and recently parallel reaction monitoring (PRM) have been demonstrated to be accurate targeted quantitation methods for both glycans and glycopeptides [178,179,180,181]. The limitation of MRM and PRM is that they cannot be used for untargeted analysis. However, with more glycomic and glycoproteomic work performed, more targets will be documented for further MRM and PRM analysis of biological samples.

#### 2.3.4. Software to Facilitate Automated Data Processing

Glycomics and glycoproteomics are focusing on hundreds to thousands of structures, causing data processing and interpretation time-consuming. The structural identification becomes even more complicated when processing glycoproteomic data since it needs the accurate assignments of both glycan and peptide structures. In addition, when considering different glycan compositions and isomers on the same peptide backbone, manually identification from biological samples such as blood serum or cell lines would be dramatically inefficient. Therefore, automated data processing software is helpful, especially for large sample cohorts. 

Common software for glycan characterization include GlycoMod [182], GlycoReSoft [183,184], Glyquest [185], SysBioWare [186], SimGlycan [187], and MultiGlycan [188]. These software employed different algorithms and databases for glycan identification. Noticeably, MultiGlycan can generate quantitative results automatically. Additionally, there are several de novo glycan identification software such as GlycoDeNovo [189], Glyco-Peakfinder [190], and Glycoforest [191]. These de novo software do not identify glycan structures based on a known library but from their MS and MS/MS patterns. In addition to the aforementioned software, there are assistant tools, such as GlycoWorkbench [192] and Skyline [193], widely used in glycomic studies. Skyline is also a powerful tool in glycoproteomic studies.

Common software for glycopeptides/glycoprotein characterization include GlycoPep ID [194], SimGlycan [187], GlycoMiner [195], GlycoPeptide Search (GPS) [196], GlypID 2 [197], GlycoPep Grader (GPG) [198], MSFragger [199], pGlyco [200], Byonic [201]. Additionally, there are more software developed by academic groups such as Glyco-Proteome Analyzer (I-GPA) [202], SweetNET [203], Glyco-DIA [204], etc. [205,206,207,208,209]. Most of the academic software are open source; however, some of them focus on specific goals, are not user-friendly, and few of them keep updating. In contrast, commercialized software Byonic, which is easy-to-use and flexible to customized search, has been the most widely used software in glycoproteomics.

Although plenty of software have been developed and applied for glycomic and glycoproteomic data processing, none of them can achieve automated isomeric assignment and quantitation, which is necessary to address in future work. However, the fact that some software can assign the same compositions to different retention times (such as MultiGlycan) indicates the potential success of further development of isomeric identification.

In general, together, these advanced techniques and software enable efficient and reliable analyses of glycomics and glycoproteomics, which have been applied to address biomedical issues, including neurodegenerative diseases.

## 3. Glycomics and Glycoproteomics of Human Biofluid

Biofluids, such as serum, plasma, or urine, are logical sources for biomarker discovery as they have been among the easiest clinical samples to obtain [210], making them ideal for investigating the expression difference of glycoproteins in longitudinal studies and during different disease stages or in healthy subjects. Aberrant glycosylation has been associated with many diseases and can be investigated from biofluidic proteins such as immunoglobulin G (IgG) [32], alpha-1-acid glycoprotein (AGP) [31], or haptoglobin [61]. In addition, most of the broadly validated cancer biomarkers are biofluidic glycoproteins, including but not limited to alpha-fetoprotein (AFP), CA19-9 (cancer antigen 19-9), CA125, CA15-3, carcinoembryonic antigen (CEA), and prostate-specific antigen (PSA) [211]. Therefore, the characterization of biofluidic glycoproteins is of great interest to better understand the attributes of glycoforms in disease development and screen glycan or glycopeptide indicators. 

The characterization of glycopeptide using the methodology mentioned above has exhibited alterations of glycopeptide expressions in biofluidic samples collected from patients [212,213]. In recent research, Zhang et al. [213] detected 134 *N*-glycopeptides from plasma/urine samples in 15 patients to demonstrate the differential expressions of glycoproteins and the ratio of fucosylated to nonfucosylated *N*-glycopeptide that could be indicators of papillary thyroid carcinoma. In another study, follicular fluid glycoproteomics of 57 participants were analyzed to reveal 10 differentially expressed glycoproteins in women with polycystic ovary syndrome (PCOS) [212]. Although many efforts have been performed to acquire deep insight into the expressions of glycopeptides in biological processes, most of the works in the recent five years were conducted on cells or tissues [214], and the number of samples was quite limited compared to glycomics analysis. The glycoproteomics analysis of bulk biofluidic samples still needs to be enhanced for a better glyco-marker investigation. 

Glycomics is another approach to study protein glycosylation. More techniques can be employed in glycomics research with higher sensitivity than the well-established glycoproteomics technique allowing a more reliable identification of glycosylation patterns due to the absence of a peptide backbone. In the last decade, numerous works have been made to investigate glycome expression changes in different biological samples, thus facilitating understanding glycans’ roles in multiple bio-functions. The number of samples (cohort) was relatively large for those biofluid-based glycomics studies because biofluids such as serum or plasma are obtainable from both patients and healthy people. Rudd and coworkers analyzed over 1000 plasma samples (including 633 colorectal cancer (CRC) patients and 478 age- and gender-matched healthy people) to identify potential glycan markers for CRC. 2-AB labeled glycans were characterized using HILIC-LC-MS. Several glycans and glycan peaks were employed to achieve the prediction of CRC with a 77.8% accuracy (with 100% specificity and 50% sensitivity). This is one of the glycomics studies that investigated the highest number of clinical samples and performed both screening and verification procedures, thus enhancing the reliability of the glycan makers discovered in this study [215]. 

Kamiyama et al. performed a serum glycomics study on 369 hepatocellular carcinoma (HCC) patients and 26 healthy people. Methyl esterification and MALDI-TOF permitted the correlation of 67 *N*-glycans to the clinical index of patients. Two glycans were identified as significant HCC recurrent and prognosis indicators through receiver operating characteristics (ROC) analysis and following correction analysis [216]. Zhao et al. investigated *N*-glycan profiles from 347 serum samples (219 CRC patients and 128 age- and sex-matched healthy people) using a DNA sequencer-assisted/fluorophore-assisted carbohydrate electrophoresis (DSA-FACE). Two *N*-glycan expression-based mathematical models were established and showed better diagnostic capacities than the FDA-approved biomarker CEA. One model was further verified in the follow-up studies and considered a better biomarker for CRC diagnosis [67]. 

Not only *N*-glycans but also *O*-glycans have been proved to be essential in cancers. [217]. In addition to *O*-glycans, glycan isomers also exhibited significant expression changes in biofluids of many cancers [120,218,219,220]. However, the cohort applied in these studies was still not large enough to draw a reliable conclusion about *O*-glycan and glycan isomer markers. 

In recent years, glycomics and glycoproteomics of human biofluids have been of great interest. This has prompted more studies in this field to better understand which role glycosylation plays in different diseases. Several additional studies have been performed and revealed that significant changes in glycans’ expression and glycopeptides’ expression are to be found when studying the biofluids of many diseases. In this review, we listed representative examples that investigated a large cohort for glycan biomarker discovery. Although glycomics and glycoproteomics analysis of human biofluids provide more information for clinical diagnosis and disease prognosis, it remains a major challenge since no glycan biomarkers have been approved by the FDA. The clinical sample cohorts for glycomics and glycoproteomics analysis are still not large enough to verify the reported potential glycan and glycopeptide markers. In addition, the analytical time needed for glycomics and glycoproteomics is long, and the technical requirements of comprehensive characterization of glycan/glycopeptide patterns are relatively high due to the complex steps in sample preparation protocols and complicated operation of LC-MS as well as the following data interpretation. Only experienced scientists can conduct such experiments and interpret data, thus preventing the routine use of these techniques in hospitals. In future studies, these challenges demand faster, simpler, more reliable glycomics and glycoproteomics strategies and larger sample cohorts.

## 4. Glycoproteomics and Neurodegeneration

### 4.1. Glycosylation and Neurodegenerative Diseases

**Figure 2:**
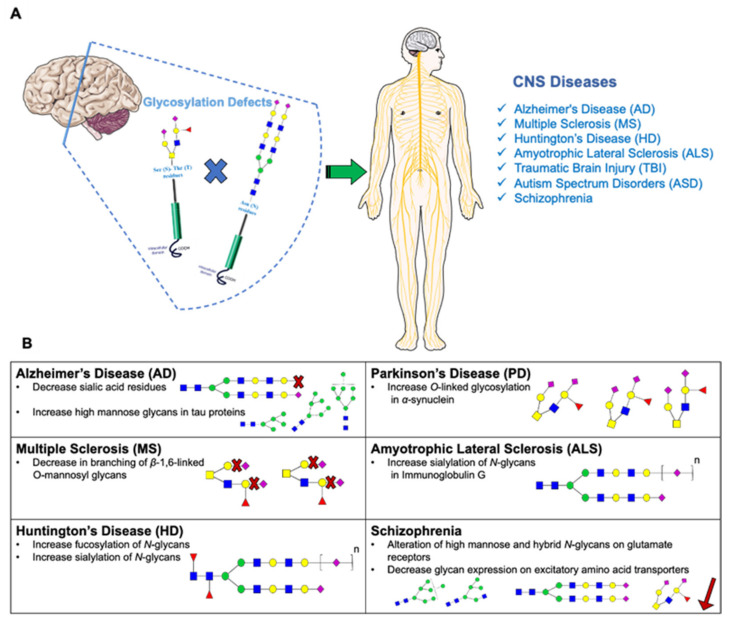
Correlation between glycosylation changes and CNS diseases. (**A**) Depiction of the consequences of glycosylation defects occurring in the different lobes of the human brain. (**B**) Characterization of consequences of altered glycosylation based on the type of neurological or psychiatric disease formed.

In the last decade, researchers worked extensively to unveil the relationships between glycoproteins and neurodegenerative diseases, including Alzheimer’s disease (AD), Parkinson’s disease (PD), Huntington disease (HD), Multiple Sclerosis (MS), and Amyotrophic Lateral Sclerosis (ALS). These relationships are depicted in Figure 2 and Table 3 found just below.

#### 4.1.1. Alzheimer’s Disease

Alzheimer’s disease (AD) is a neurodegenerative disease mainly related to the accumulation of amyloid-*β* (A*β*) peptides in the brain. In the study of Hüttenrauch et al., they discovered that Glycoprotein Nonmetastatic Melanoma Protein B (GPNMB) is a novel AD-related factor not only in transgenic mice models but also in sporadic AD patients. In transgenic AD models, their immunohistochemistry, ELISA, and expression profiling tests found that GPNMB increases in an age-dependent manner and is co-localized with IBA1-positive microglia cells that cluster near amyloid plaques in the brain. However, GPNMB is increased in cerebrospinal fluid (CSF) and brain samples in sporadic AD patients, whereas normal values of GPNMB are found in non-demented controls [238]. 

Some researchers like Ilic et al. were interested in investigating the relationship between the hippocampal expression of brain-specific neuroplastin isoform (Np65) and tau pathology in AD. Np65 expression and localization were analyzed in 6 human hippocampi with confirmed AD neuropathology and compared to six age- and gender-matched control hippocampi by immunohistochemistry measurements. Their results suggest that this glycoprotein is involved in tissue reorganization and can represent a potential molecular marker of plasticity response in the early neurodegeneration process of AD [239]. However, García-Ayllón et al. were interested in knowing the regulation of human natural killer-1 (HNK-1) in neurodegenerative diseases, especially in AD, because it was not well elucidated. This study showed that HNK-1 is decreased in the brain of AD since it is influenced by the *β*-amyloid protein formation [240].

Several other studies tested the effect of *P*-glycoprotein on amyloid clearance. This glycoprotein is located across the blood-brain barrier, and it is the efflux transporter that is highly expressed on the luminal side, supporting the process of A*β* clearance from the brain [241,242]. These studies concluded that P-glycoprotein is considered a novel pharmacological target in AD [243], which plays a crucial role in the clearance of amyloid-*β* 42, and amyloid-*β* 40 [242,244]. 

#### 4.1.2. Huntington’s Disease

Huntington’s disease (HD) is a fatal genetic neurodegenerative disease that is directly related to the aggregation of mutant huntingtin (HTT) protein where the expansion of polyglutamine occurs [213,245,246]. According to the literature, different glycoproteins are associated with this disease [247,248]. Still, the number of studies that tackled this topic for HD is considered small compared to other neurodegenerative diseases. In the 2015 study of Kao et al., they discovered that higher levels of P-glycoprotein were observed in the brain capillaries of human HD patients. Their results also showed that R6/2 HD transgenic mice with the human mutant HTT gene had enhanced NF-kB activity in their brain capillaries [245]. Thus, they concluded that mutant huntingtin caused a change in the expression of *P*-glycoprotein through the NF-kB pathway in brain capillaries in Huntington’s disease patients and altered the availability of P-glycoprotein substrates in the brain.

Another study suggests that the expression of microRNA miR-27a is associated with HD. In this study, they used an in vitro HD mouse model to check the effect of miR-27a on mutant huntingtin (HTT) aggregation. Their immunocytochemistry tests showed that mutant huntingtin (HTT) aggregation was elevated with differentiation, and they examined the phenotype of HD after transfecting miR-27a in the R6/2-derived differentiated NSCs [246]. However, several old studies aimed to check the glycoproteins associated with different neurodegenerative disorders and not only HD. These studies showed that Huntington’s disease is linked to the expression of the histocompatibility glycoprotein HLA-DR [249], p53, and CREB-binding protein where it represses their transcription in a transgenic mouse model of HD [250].

#### 4.1.3. Multiple Sclerosis Disease

Multiple sclerosis (MS) is another neurodegenerative disease mainly caused by demyelinating the human central nervous system (CNS). Almost all of the studies in the literature studied the relationship between MS disease and the expression of myelin oligodendrocyte glycoprotein (MOG) because the role of pathological auto-antibodies against the latter in MS disease models is highly controversial [251]. These glycoproteins are located on the myelin sheath’s outer external surface, insulating lipid layer around neurons [252]. MOG is considered a possible target antigen for antibodies in MS disease models and other demyelinating diseases [253]. 

The study of Khare et al. showed that antibodies derived from adult MS patients exacerbate experimental autoimmune encephalomyelitis (EAE) in ‘humanized’ mice that transgenically express human FcγRs (hFcγRs). Additionally, this exacerbation is primarily dependent on MOG recognition by the human-derived antibodies, and enhancing the affinities of these antibodies for specific FcγRs demonstrates that FcγRIIA is more important than FcγRIIIA in mediating disease exacerbation. Thus, this study showed the relationship of the contribution of MOG-specific antibodies to MS and unveiled internal mechanisms that could help the development of new therapeutic targets [253].

Another study analyzed T-cells’ reactivity and the related frequency to utilize a novel technique to detect any antigen-specific T-cells with bead-bound MOG as a stimulant. They tested samples of peripheral blood mononuclear cells from natalizumab-treated persons with MS versus healthy people, and these samples were analyzed using IFNγ/IL-22/IL-17A FluoroSpot. The results of these studies revealed a more significant number of IFNγ, IL-22, IL-17A along with double and triple cytokine producing MOG-specific T-cells in MS patients compared to the control sample. Additionally, their data showed that more than 50% of MS patients have MOG-specific T-cells, which gives an insight into the link between this glycoprotein and MS disease [254].

#### 4.1.4. Amyotrophic Lateral Sclerosis Disease

Amyotrophic lateral sclerosis (ALS) is a neurodegenerative disease affecting motor neurons, leading to cognitive and physical impairments [255]. Additionally, available therapeutic options cannot slow the progression of these diseases, and novel treatments are urgently needed. In the research of Budge et al., they studied the association between inflammatory cytokines such as interleukin-6 (IL-6), interleukin-1*β* (IL-1*β*), and tumor necrosis factor-α (TNF-α) and ALS as well as several neurodegenerative diseases. This study revealed that nonmetastatic glycoprotein melanoma protein B (GPNMB) is neuroprotective in an animal model of ALS. Future studies should investigate the more potential therapeutic value of GPNMB in ALS [255].

However, P-glycoprotein is another glycoprotein found appealing in ALS models in several recent studies. In the paper of Chan et al., they studied the critical obstacles for drug delivery residing at the level of both the blood-brain barrier (BBB) and the blood-spinal cord barrier (BSCB). Such obstacles that would limit the efficacy of therapeutic agents were P-glycoprotein (P-gp), breast cancer resistance protein (BCRP), and multidrug resistance-associated protein 2 (MRP2). They checked their expression in the ALS SOD1-G93A transgenic rat model across the three stages of disease progression: pre-onset, onset, and symptomatic. In the symptomatic stage, their results showed an increase in both P-glycoprotein transport activity and expression compared to the control sample, while no change in the animals with BCRP and MRP2. Therefore, their experiments and immunohistochemical analysis in the brain and spinal cord capillaries of SOD rats suggested that any treatment should not be from P-glycoproteins substrates to improve therapeutic efficacy in the CNS during ALS progression [256].

Another recent study showed that the upregulation of P-glycoprotein affected by disease advancement progressively reduces central nervous system penetration and therapeutic efficacy of the ALS-related drugs. Additionally, they discovered that glutamate, which is abnormally secreted by mutant SOD1 and sporadic ALS astrocytes, would enhance the upregulation of P-glycoprotein expression in endothelial cells by activating NMDA receptors. However, not all ALS forms worked with the same mechanism since C9orf72-ALS astrocytes did not affect endothelial cell P-glycoprotein expression. Therefore, their results unveiled the complex molecular interplay between astrocytes of different ALS forms and endothelial cells potentially occurring in disease affecting the progression of ALS disease and the efficacy of pharmacotherapies [257].

#### 4.1.5. Parkinson’s Disease

Parkinson’s disease (PD) is a neurodegenerative disorder affecting dopaminergic neurons in the brain and especially the substantia nigra (SN). This disease causes stiffness and abnormal muscle movements. Several relationships between PD and glycoproteins have been studied in the last five years, and they are helping discover novel therapeutic candidates to treat PD patients. In the paper of Dunn et al., the researchers discovered the involvement of synaptic vesicle glycoprotein 2 (SV2) in PD through modifying the sensitivity of L-DOPA and the nicotine of neuroprotection genetically. Their results also showed that SV2C expression is predominantly changed in postmortem brain tissue from mice PD samples but not in other neurodegenerative diseases such as Alzheimer’s disease or multiple system atrophy. Therefore, they suggested that SV2C disruption is a distinctive characteristic of PD that likely leads to dopaminergic dysfunction in the neurons [258].

GPNMB is another glycoprotein linked to an increased risk of PD, as stated by Moloney et al. In this study, alterations in the level of GPNMB were observed in the SN part of the brain in PD cases compared to stable levels in age-matched controls. However, the transgenic mice modeling synucleinopathy experiments demonstrated an increase in GPNMB or glucocerebrosidase (GCase) deficiency compared to wild-type mice. Thus, the expression of GPNMB in SN of PD cases and the induction of GPNMB after experimental glycosphingolipid increases are considered the potential for primary lipid-induced degeneration in PD [259].

Moreover, in the study of Gan et al., they created microRNA (miR)-124-loaded rabies virus glycoprotein (RVG) 29 surface-conjugated polymeric nanoparticles (NPs) that treat neuroinflammation in PD because they assumed that elevation in the intracellular concentration of miR-124 would affect the prognosis for PD. Moreover, their immunohistochemical staining results showed that exogenous delivery of these nanoparticles downregulated MEKK3 expression in animal studies. Therefore, this study revealed that miR-NPs could inhibit pro-inflammatory signaling and improve neuroprotection in Parkinson’s disease patients [260].

Protein glycosylation contributes to the pathogenesis of different human diseases like PD, and various studies tackled this issue. A recently published study identified alterations in glycosylation in a mouse PD model using biotinylated Agaricus bisporus lectin after injecting these models with 1-methyl-4-phenyl-1,2,3,6-tetrahydropyridine. The data analysis from lectin affinity chromatography coupled with mass spectrometry showed a significant increase in the glycosylation of microtubule-associated protein 6 in PD mice compared to control mice. This research showed crucial innovative information, such as the association between hyperglycosylated MAP6 and the pathogenesis of PD [261].

Finally, myelin-associated glycoprotein (MAG) was also studied along with its relationship with PD in the study of Papuć et al. They measured IgM autoantibodies against MAG using ELISA for 132 subjects (50% are PD patients). This study demonstrated an elevation in the production of anti-MAG IgM antibodies in PD patients, along with an activation of the humoral response against MAG in Parkinson’s patients [262]. However, the role of anti-MAG antibodies as biomarkers of PD is not clear, and further studies are warranted.

### 4.2. Glycoproteomics and Psychiatric Disorders

#### 4.2.1. Depressive Disorders

All studies of glycomics and mood disorders focused on the major depressive disorder (MDD) and hinted towards the implication of *N*-glycosylation in the etiology of MDD. The first clinical study compared the protein glycosylation pattern in depressed and remitted patients with MDD [263]. Blood samples were collected from ten patients with MDD (defined as Hamilton Depression Rating Scale > 18) during both depression and remission states and ten healthy controls. Binding signals for ten lectins were significantly altered in the remitted MDD group compared to the depressed MDD and control groups, suggesting that these lectins may be stress response markers in MDD. These changes were most significant for Trichosanthes Japonica Agglutinin I (TJA-I), Sambucus Nigra Agglutinin (SNA), Griffonia Simplicifolia Lectin I Isolectin B4 (GSL-I-B4), and Helix Pomatia Agglutinin (HPA) (*p* < 0.001). Additionally, analysis of the expression levels of sialyltransferases in leukocytes of participants showed that the expression of ST6GALNAC2 was significantly decreased in remitted patients compared to depressed patients [263]. As sialylation is thought to influence brain structure and function [264], alteration in glycoproteins’ sialylation can act as a biomarker of interest in MDD [263].

In an exploratory study, Park et al. assessed the *N*-glycan profiles of 18 individuals with MDD at baseline and six weeks after antidepressant treatment. Results showed gender-dependent correlations with the severity of depressive symptoms before and after initiating antidepressant treatment. Indeed, at T0, 11 glycan peaks were significantly correlated with the Hamilton Depression Rating Scale (HDRS) score in all patients, with differential profiles of correlation in males and females. Additionally, only females had 2 IgG4 *N*-glycan glycoforms-containing bisecting *N*-acetylglucosamine—significantly correlated with the T0 HDRS score. After six weeks of antidepressant response, all cohorts showed no difference in glycan peak level or IgG *N*-glycan profiles between responder and non-responder groups, both at T0 and T6. However, in females, oligo-mannose *N*-glycan levels and 3 IgG4 *N*-glycosylation traits were different between responder and non-responder patients at T0. The authors concluded that specific glycosylation traits might be associated with MDD severity and antidepressant response in a gender-dependent fashion [265]. 

Lastly, Boeck et al. compared the serum *N*-glycan profiles of 21 females with an acute depressive episode to 21 non-depressed healthy females. Compared to controls, women with MDD showed significant alterations in the serum levels of several *N*-glycan structures: reduced total level of agalactosylated *N*-glycans, particularly two agalacto core-α-1,6-fucosylated biantennary glycans (NG1A2F), and increased total level of triantennary *N*-glycans, mainly the branching α-1,3-fucosylated triantennary glycan (NA3FB) and the non-fucosylated biantennary glycan (NA2). These differences significantly correlated with depressive symptom severity, and to a lesser extent, with interleukin-6 levels and C-reactive protein levels. Additionally, post-hoc analyses revealed that the alterations in *N*-glycan profiles were most pronounced in MDD patients with a history of childhood sexual abuse [266]. 

Overall, the results of these studies point towards alterations in the serum *N*-glycan profile in patients with MDD that are potentially linked to inflammatory processes and that might be used as future biomarkers for diagnostic evaluation and assessment of treatment response. 

#### 4.2.2. Neurodevelopmental Disorders

Zwaag et al. performed genome-wide copy number variant analysis of the DNA of 105 patients with autism and 267 healthy controls. This was followed by an independent analysis of autism chromosomal susceptibility loci derived from the literature to see whether glycobiology-related genes are commonly present in regions that confer risk for autism. The authors identified seven cytogenetic regions from their participants and selected six susceptibility loci identified from previous genetic linkage analysis studies [267]. Gene-network analysis of the 13 susceptibility loci revealed an overrepresentation of genes related to glycobiology. Indeed, six genes (B3GALNT2, B3GALT1, GAL3ST2, B3GNT5, GALNTL5, and ARSA) involved in glycobiology were highly ranked in the loci and many of which are expressed in developing murine brain regions known to be altered in the human autistic brain. The authors suggested that dosage alterations, via genomic losses and gains, in these genes contribute to dysfunction in glycosylation pathways, and interaction with other culprit factors would produce an autism phenotype [267]. 

In another study by Pivac et al., components of the plasma *N*-glycome were quantified in 81 children and 5 adults with autism, 99 children with attention-deficit hyperactivity disorder (ADHD), and 340 matching healthy controls. The study did not find any differences in plasma glycans of participants with autism compared to those of healthy controls. However, several highly significant associations were observed in individuals with ADHD. The most notable changes in plasma glycans in the ADHD group were increased glycan groups GP11 and DG7 and a decrease in GP12 (*p* < 0.001). Furthermore, ADHD was associated with a regular pattern of changes in GP11, GP12, GP14, GP16, tetrasialoglycans, and trigalactosylated glycans. The glycans increased in ADHD were composed of biantennary glycans and antennary fucosylation (A2FG2), whereas those that were decreased consisted of tri- and tetra-antennary glycans [268]. Future studies should further assess the glyco-phenotype of individuals with neurodevelopmental disorders, as this might shed light on the pathophysiology of these conditions and open a new direction or the development of potential therapeutics. 

#### 4.2.3. Schizophrenia and Related Psychotic Disorders

A growing body of clinical research has reported glycosylation and glycomic abnormalities in patients with schizophrenia [269]. These studies are summarized in Table 4 inserted right below. Findings suggested that dysfunction in glycobiology pathways could contribute to the pathophysiology of schizophrenia and hold potential as diagnostic and treatment tools for the disease. 

#### 4.2.4. Sleep-Wake Disorders

Only one study looked at glycomics of sleep disorders, particularly rapid eye movement sleep behavior disorder (RBD). In this study comparing serum glycomes of nine patients with RBD to ten healthy controls, Dong et al. identified 56 *N*-glycans in the RBD group compared to 59 *N*-glycan structures in healthy controls. On average, 60% were sialylated structures, 20% fucosylated structures, and 20% high mannose structures. A total of 16 *N*-glycans were found to be significantly altered in the RBD group (*p* < 0.05), of which six were overexpressed. *N*-glycans with the composition of HexNAc4Hex5Fuc1, HexNAc5Hex5, and HexNAc4Hex5Fuc1NeuAc1 displayed the most substantial difference between the RBD group and healthy controls (*p* < 0.01). Moreover, HexNAc4Hex5Fuc1NeuAc1 showed a relatively high abundance (4 ± 3% in the RBD group vs. 3.1 ± 0.7% in healthy controls). Alternatively, 7 *N*-glycan isomers were significantly different between the two groups (*p* < 0.05), of which HexNAc4Hex5Fuc1NeuAc1 (4511-2) and HexNAc4Hex5Fuc1 NeuAc2 (4512-2) showed the most substantial difference (*p* < 0.001) with higher levels in the RBD group than in healthy controls. The authors concluded that the differentially expressed *N*-glycans in the RBD group could be potential diagnostic biomarker candidates that will provide further insight into the neurodegenerative processes commonly observed in patients with idiopathic RBD [123]. 

#### 4.2.5. Trauma- and Stressor-Related Disorders

To investigate whether traumatic stress accelerates physiological aging, Moreno-Villanueva et al. analyzed the *N*-glycosylation profile in 13 patients with post-traumatic stress disorder (PTSD), 9 high-stress trauma-exposed individuals, and 10 low-stress healthy controls. Although the study did not find significant differences in plasma *N*-glycans between the three groups, results suggested that cumulative exposure to traumatic events advances the aging process. Indeed, patients with PTSD and high stress had significantly higher values on the GlycoAge Test compared to controls (*p* = 0.03), equivalent to an acceleration of their aging by 15 years. The traumatic load was positively correlated with the GlycoAge Test (*p* = 0.02), while gender did not affect it [280]. 

In another study looking for PTSD biomarkers, *N*-glycomic profiles of 299 male veterans with PTSD were compared to 244 healthy controls [281]. Results showed that six plasma *N*-glycans were significantly altered in patients with PTSD compared to controls. Among these plasma *N*-glycans, four (GP14 = A2G2S1, GP27 = A3G3S3, GP33 = A4G4S3, GP39 = A4F1G4S4) were significantly higher, whereas two (GP16 = FA2G2S1, GP19 = M9) were significantly lower. The severity of PTSD was not associated with different plasma *N*-glycans, and IgG *N*-glycans were similar between groups. In this study, patients with PTSD did not show signs of accelerated physiological aging on the GlycoAge Test compared to controls [281].

There are also few reports of observed differences in the serum glycosylation profile of highly stressed individuals. This includes significantly higher concentrations of 57 kDa glycoprotein in war prisoners [282] and soldiers [283] and higher concentrations of *N*-oligosaccharides, mostly sialic acid in soldiers [284] as compared to healthy controls. The above findings of altered glycome profiles in individuals with acute stress or PTSD suggest that trauma-related disorders might be mediated by changes in glycosylation patterns [285].

## 5. Glycoproteomics and TBI

### 5.1. Post-Translational Modifications and TBI

**Figure 3:**
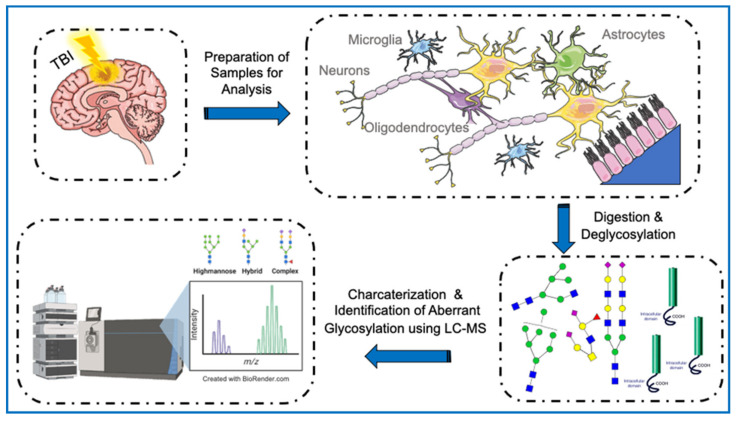
An overview of the after-effects of TBI on the neurological components of the brain, ultimately leading to aberrant glycosylation as shown by MS-based glycoproteomics. TBI: traumatic brain injury, MS: mass spectrometry.

After mechanical insults, the brain acquires direct irreversible characterizations causing both focal and diffuse injury. As a consequence, TBI warrants a progressive cascade of cellular, neurochemical, and metabolic events that endorse the disruption of normal brain function and modulate gene expression, ultimately leading to the exacerbation of neural injury [286], as shown in Figure 3 inserted below. The primary and secondary injury conditions accompanying TBI have been proven to modify cellular PTM profiles and increase the risk of developing neurodegenerative diseases such as AD and PD. Shifts in PTM physiology may lead to health and disease homeostasis fluctuations, affecting cell signaling pathways and protein interactions and triggering several dysfunctional neurological manifestations [287]. Researchers have utilized gene knockout studies in mice to show that certain PTMs are indispensable for neural development. The importance of PTMs may stem from their major participation in the biosynthesis of specific neuronal components, where aberrant progressions may lead to alterations of normal neural development and migration [288]. In a recent study, Endo demonstrated that aberrant mannosylation during development could alter neuronal migration, triggering several congenital disorders such as muscular dystrophy [289]. 

Glycoproteomic analysis has been employed in a limited number of studies to expose the crucial roles of PTM alterations in the progression of TBI pathology. In 2016, Yang et al. conducted a study on 224 TBI patients investigating the role of a specific aberrant PTM in neuronal damage following TBI [290]. The study elucidated the involvement of tau phosphorylation in subsequent cognitive impairment, altering the normal PTM profile of patients with variable TBI severity. Results showed a direct correlation between tau phosphorylation and the modifications closely related to neuronal damage. In parallel with the increase of TBI severity, rapid inductions of tau phosphorylation were found at the focal injury sites. This was accompanied by the enhanced roles of GSK-3*β* and PP2A, being key participants in the process of tau hyper-phosphorylation as TBI severity increases [290]. Therefore, tau pathology may prove to be a primary target for TBI therapy due to the significant impact on cognitive function and neurotransmission. 

In addition, protein carbonylation is considered a well-documented irreversible PTM that leads to the loss of protein function. Carbonylation is generally a consequence of oxidative stress seen in many neuropathologies such as AD and PD. A 2014 study used the brains of adult Sprague Dawley rats to visualize protein carbonylation after a controlled cortical impact (CCI) [291]. Immunohistochemistry results showed that this TBI model led to the co-localization of carbonylation cell markers in astrocytes, neurons, microglia, and oligodendrocytes of the ventral portion of the dorsal third ventricle and the lining above the median eminence. The further proteomics analysis determined that the proteins most affected by carbonylation following TBI were dihydro pyrimidase-related protein 2, glial fibrillary acidic protein, fructose-bisphosphate aldolase A, and fructose-bisphosphate aldolase C [291]. Another study considered the effects of TBI on cytoskeletal proteins present in PC12 cells [292]. Results indicated the increase of oxidative stress in these cells as well as the increase of carbonylation in *β*-actin and *β*-tubulin. This suggested that the manifestation of TBI may lead to carbonylation of cytoskeletal proteins, which in turn undermines their stability.

### 5.2. Glycosylation in Neurotrauma

Glycosylation is a ubiquitous PTM responsible for most protein modifications in human cells and organisms. Glycan binding proteins such as lectins have been employed to visualize and identify glycan structures in CNS trauma. Glycan biomarkers discovered through quantitative glycomics such as CA125 and CEA have been proven to improve the clinical prognosis and diagnosis of neurotrauma, attaining a diagnostic capacity and prompting the improvement of glycomic techniques [293]. In the CNS, eukaryotic cells like neurons and glia are crusted with a layer of glycans, functioning dynamically to allow cellular communication. Different cell types arise from highly proliferative neural stem cells via variable expressions of glycan-rich molecules. The association of glycan structures with other entities paves the road for neural development, cellular differentiation, and normal molecular trafficking during neural development [3]. Any disruption or alteration occurring in the normal glycosylation profile can lead to disastrous neurological outcomes. Aberrant glycosylation, such as elongation or trimming of glycan structures, can be linked to many neurological problems, disorders, and immune responses. For example, alterations in the expression of stage-specific embryonic antigen-1 (SSEA1) and tumor rejection antigens (TRA), which are glycan antigen conjugates, can lead to aberrant differentiation of neural stem cells into mature neurons or glial cells [9,280].

Within the ER-Golgi network, there exist eight major pathways of glycan generation. The initiation and extension of glycan chains depend on pathway-specific glycosyl transferases, but it can also rely on transferases that serve a number of different pathways. Mutations occurring in any of the main players of these pathways may lead to neurological deficits. In *N*-linked glycosylation, the TUSC3 gene encodes an oligosaccharyltransferase subunit, enhancing glycosylation efficiency by slowing down the process of glycoprotein folding. Mutations or deficiencies in the TUSC3 gene contribute to the decrease of total and intracellular magnesium levels in mammals, possibly permitting the manifestation of non-syndromic intellectual disability [294]. Other transfigurations affecting CNS proteins include the misfolding of the cellular prion protein (PrP^C^), a glycosyl-phosphatidyl-inositol (GPI) anchored glycoprotein. Misfolding PrP^C^ transforms it into its pathogenic counterpart PrP^Sc^, allowing its aggregation through altered interactions with proteins and lipid membrane components and subsequently spreading the pathology in prion diseases. Moreover, glycosylation deficiencies heighten human PrP cytotoxicity, enhancing the association with higher levels of reactive oxygen species (ROS), which leads to an increase in oxidative stress [295].

In neurotrauma, the role of glycosylation has been illustrated through spinal cord injuries (SCI). The pathology of SCI, as for TBI, is mediated by the increased release of proinflammatory cytokines during the secondary injury conditions, leading to axonal destruction, demyelination, and neuronal loss. Following SCI, a sialic acid molecule may be added to the membrane proteins of ion channels, altering neuronal ion conductance and possibly inducing apoptosis of neurons and oligodendrocytes. Membrane proteins can also undergo excessive glycosylation, exacerbating the excitotoxic environment that spreads after trauma [296]. Li et al. found higher concentrations of glycosylated proteins in the dorsal root ganglion neurons, associated with possible sialylation on voltage-gated channels. Gene knockout studies performed on mice illustrated the role of glycosyltransferase enzymes following trauma, proving that the biosynthesis of glycans has a primary impact on neuronal development and glial scar formation. Aberrant glycosylation and *O*-mannosylation may further alter normal neural migration, increasing the pathology of trauma [297].

### 5.3. Neuronal Death following Experimental TBI

TBI is a pathological event triggering neuropathological conditions. As primary insult occurs, the direct force trauma is limited to specific underlying tissue. This mechanical hit may result in acute hemorrhage, neuronal loss, and necrotic cell death depending on TBI severity. Secondary injury conditions favor diffuse and long-lasting damage that targets both glia and neurons. The secondary insult of TBI may provoke a delayed form of cell death, allowing progressive neurodegeneration and damage site expansion [298]. Neuroinflammation is considered the main player in the secondary phase of TBI pathology, depending on the release of proinflammatory cytokines after the initial trauma. The activation of inflammasome complexes is an essential step of neuroinflammation, subsequently triggering a stage of neuronal death called pyroptosis. For example, inflammasome complexes may be involved in activating caspase-1, catalyzing the cleavage of interleukins such as interleukin-18 (IL-18) and IL-1*β* into their active forms as pro-inflammatory cytokines [299]. Within the CNS, microglia function as the primary intermediaries of the innate immune response, having a dual beneficial and detrimental role that results in tissue repair or neurodegeneration, respectively. On the one hand, microglial cells remove cellular debris formed after impact and release anti-inflammatory cytokines that prevent further neuronal injury. In addition, the activation of highly reactive microglia may result in the release of cytotoxic pro-inflammatory mediators that inhibit the restoration of cellular integrity and contribute to neuronal dysfunction and death [300].

An important process in secondary damage following TBI is excitotoxicity [301]. It occurs mainly due to the excessive activation of the excitatory amino acid (EAA) receptors, where the *N*-methyl-*D*-aspartate (NMDA) receptors play the most prominent role [302]. In moderate and severe TBI, mechanical insults damage the protein channels and disturb the ionic homeostasis [303]. The high quantities of glutamate bound at the NMDA receptor promote a considerable Ca^2+^ influx causing a Ca^2+^ overload, which leads to an ionic imbalance where the increase of sodium (Na^+^) influx and potassium (K^+^) efflux lead to additional depolarization. Excitotoxicity manifested as the excessive depolarization of neurons, glia, and cerebral endothelial cells will occur. The latter will lead to neuronal destruction, cell death, and dysfunction, ultimately driving the cell toward oxidative stress [304,305,306].

Once excitotoxicity occurs, the excess of Ca^2+^ could promote the production of ROS as well as nitric oxide (NO), where protective mechanisms such as antioxidants fail to control free radicals [307]. The outcome is oxidative stress. The latter can be defined as an impairment inflicted by ROS production and its detrimental consequences on proteins, lipids, and DNA [308]. An aspect of TBI is the cellular damage enabled by the oxidation of both lipids and proteins, where TBI severity can be correlated with the degree of ROS-related tissue damage [309]. Oxidative stress in TBI is prominently manifested as lipid peroxidation of neuronal, glial, and vascular cell membranes as well as myelin [310]. Brain tissue is tremendously vulnerable to oxidative damage due to its high degree of oxidative metabolic activity, relatively low antioxidant capacity, and low repair mechanism activity since the neurons possess a non-replicating nature [308,311]. ROS can be produced via the arachidonic acid cascade activity, mitochondrial leakage, catecholamine oxidation, and by neutrophils [310,312]. 

The process of sequestering Ca^2+^ to the mitochondria could also lead to cell death either directly by apoptosis or indirectly through the loss of oxidative phosphorylation and failed production of adenosine triphosphate (ATP). A Ca^2+^ overload could play a leading role in the mitochondrial cytochrome c release, caspase activation, and apoptosis [313,314]. In the model of closed-head injury, mitochondrial dysfunction due to the diffuse TBI is correlated with TBI severity and measured by ATP and n-acetyl aspartate reductions [315]. The consequences of oxidative stress thus overwhelm the CNS, leaving it vulnerable to other harmful outcomes.

## 6. Glycoproteomics and Glycosylation: Role in Personalized Medicine

Personalized medicine strives to deliver customized diagnosis and treatment for patients based on a broad spectrum of parameters such as medical history, physiological and genetic status, as well as molecular characteristics [316]. PTMs like glycosylation present potential targets for disease hallmarks to be utilized in individualized medicine as they are shown to have value in diagnosis, prognosis, and therapy response [286]. This ranges from identifying genetic polymorphisms in glycosyltransferases to predict H. *pylori* infection susceptibility [317,318] to detecting glycosylation patterns in cancer characterization [319,320,321]. 

### 6.1. Glycomics and Glycoproteomics in Cancer Studies

Malignant events in epithelial cells release glycoproteins with altered glycans into the bloodstream [322], along with the increase in fucosylation and sialylation that has been observed in several carcinomas [321,323,324]. For example, high levels of carcinoembryonic antigen (CEA), a highly *N*-glycosylated glycoprotein involved in cell adhesion, have been allocated a prognostic value in colorectal cancer, indicating progressed disease stages and a well-differentiated tumor [325,326,327,328]. 

Additionally, the metastatic occurrence has been correlated with increased glycosylation in invasive tissue compared to primary carcinomas [329,330]. The upregulation of sialic acid sugars attached to glycoproteins and glycolipids has become a hallmark of several tumor cell types [331]. As metastasis and invasion depend on extracellular matrix (ECM) molecules like ECM cytokines, growth factors, and cell surface proteins, their altered glycosylation has been shown to induce contact-dependent mechanisms that allow tumor cell extravasation [332]. Specifically, gliomas, which are challenging due to their invasiveness [333], bind hyaluronic acid-based ECM to interact with the lectican family chondroitin sulfate proteoglycans and CD44 [334,335] that are involved in tumor migration. Another protein that was shown to be upregulated in invasive gliomas is brevican protein [336,337]. Vipiano et al. identified two novel isoforms of the brevican protein present in gliomas, each with a different pattern of glycosylation, and posed a role for them as diagnostic markers, as well as potential targets for immunotherapy [338]. 

Enzymes involved in PTMS are also involved in the metastatic phenotype in brain cancer [339]. GnT-V, a glycosyltransferase encoded by the gene MGAT5, was shown to be increased in brain cancer, contributing to a decrease in cellular adhesion and promoting metastasis [329,340,341]. Moreover, sialic acid epitopes allow cancerous cells to avoid immune response by hindering their recognition [342]. Hudak et al. mimicked cancer-associated sialylation by inserting sialylated glycopolymers into cancer cells’ membrane, inducing the localization of Siglecs, which are sialic acid-binding proteins on immune cells and increasing SHP-1 and SHP-2 phosphatase recruitment [343]. This resulted in NK cells’ failure to be activated against tumor cells, revealing the role of sialylation in immune evasion. 

### 6.2. Glycomics and Glycoproteomics in Prion Disease

**Figure 4:**
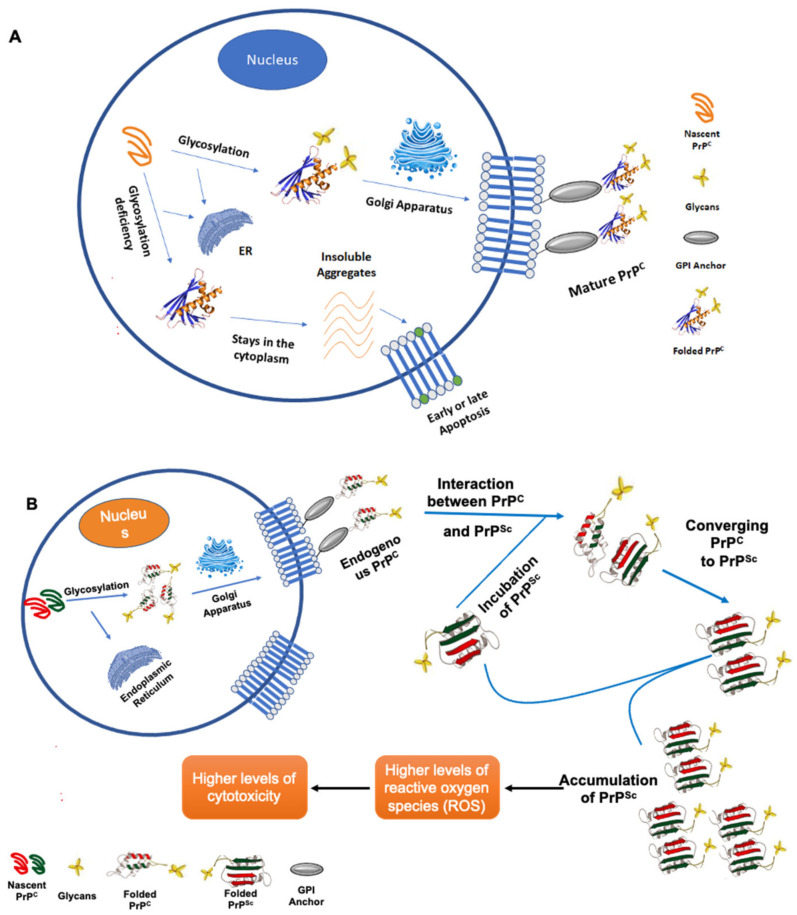
The process of glycosylation of normal PrP and the effect of deficiency in glycosylation or conversion of PrP^C^ to PrP^Sc^. (**A**) In normal conditions, nascent PrP undergo glycosylation which occurs in the Endoplasmic Reticulum (ER), then it matures in the Golgi apparatus and eventually reaches the plasma with the aid of GPI anchor. However, when glycosylation deficiency occurs, the nascent PrP becomes insoluble aggregates which leads to early or late apoptosis (green label). (**B**) Mature PrP^C^ at the level of the plasma may interact with PrP^Sc^, and this would lead to a conversion and accumulation of PrP^Sc^ which in turn would increase the level of cytotoxicity due to the presence of this prions disease.

Glycosylation also interplays with other factors that contribute to several brain-related diseases like prion disease and other neurodegenerative disorders, as shown in Figure 4. Prion disease comprises the structural change of a specific prion protein PrP^c^ into its disease-associated isoform PrP^Sc^ [344]. PrP^C^ undergoes two PTMs, the first is the attachment of GPI anchor to its C-terminal residue Ser-231, and the other is glycosylation at residues Asn-181 and Asn197 [345]. Studies have demonstrated that when PrP^C^ is unglycosylated at its *N*-terminus, it becomes more susceptible to conversion to PrP^Sc^ [346]. This was observed in a study that used murine neuroblastoma cells treated with tunicamycin, which blocks *N*-glycosylation, to reveal that reduced glycosylation of PrP^C^ predisposed it to turn into PrP^Sc^ [347]. Specifically, the immature high-mannose form of PrP^C^, which is yet to undergo complex glycosylation, is the most susceptible to PrP^Sc^ conversion. For this reason, it was postulated that mechanisms dependent on the binding of PrP^C^ to F-box-only protein Fbox2, which binds *N*-linked high mannose oligosaccharides and aids in substrate recognition by SCF complex, might play a key role in prion disease pathology [348].

The glycosylation at the *N*-terminus can hold up to five sialic acid residues [349]. These have been shown to alter the properties of the protein and play a role in the infectivity rate of the misfolded isoform PrP^Sc^ [345]. Due to the fact that sialic acid is negatively charged and is directed outwards to create a dense negative cloud [350,351], Katorcha et al. proposed that it might impose an electrostatic hindrance for PrP^Sc^ replication. Trying to prove that, the research team showed that the level of deglycosylation in the PrP^Sc^ form was less than that in the PrP^C^. Addtitionally, among some mouse strains tested in the same study, partial desialylation caused an increase in the replication rate of the protein, adding evidence that this type of PTM forms a barrier to replication. To add to this, it was observed that changes in the sialylation levels of PrP^C^ affected the formation of three different glycoforms [345] that were shown in previous studies to give rise to PrP^Sc^ in a selective manner [352]. These were later used for strain typing of the different prion subtypes [353,354]. In another study, the role of glycosylation in the subcellular localization of PrP^C^ was investigated [228] and was shown to exhibit an impaired localization at the plasma membrane. Not only this, but the study also showed that glycosylation enhances the protein’s proteinase K resistance and aggregation ability, increases ROS levels, and increases cytotoxicity.

### 6.3. Glycomics and Glycoproteomics in Neurodegenerative Diseases

Neurodegenerative diseases have also been strongly linked to changes in expression levels of glycosyltransferases [355]. In this regard, two main glycosyltransferase mechanisms are at play: ganglioside synthesis, primarily composed of sialic acid-containing glycosphingolipids, and *O*-linked *β*-*N*-acetylglucosamine (*O*-GlcNAcylation) to proteins [356]. Gangliosides, which are abundant in neuronal and glial cells [357], play a role in cell signaling, and their altered levels have been associated with amyotrophic lateral sclerosis (ALS) [358], Parkinson’s [359], and Alzheimer’s [360]. Similarly, *O*-GlcNAcylation, which has a role in synaptic and axonal function, has been linked with the same abovementioned diseases when it is significantly reduced in cells. 

Some genetic factors affecting these two mechanisms have been described to contribute to neurodegeneration. For example, recent research has shown that mutations close to the substrate-binding site of glycosyltransferase 8 domain-containing 1 (GLT8D1) causes aberrations in enzyme activity and are linked to familial ALS [361]. This study by Cooper et al. is the first to demonstrate that a disruption in the function of a glycosyltransferase is enough to cause a neurodegenerative disease [361]. Additionally, the two glycosyltransferases B3GALt4 and ST3GAL2 in neuromelanin-containing neurons in the substantia nigra show a decreased expression in Parkinson’s diseases [362]. Consistently, GM1 ganglioside-expressing cells show a similar reduced pattern [359], along with other brain gangliosides GD1a, GD1b, and GT1b, in patients with the same disease [363]. Likewise, in humans and the R6/1 mouse model of Huntington’s disease a decreased expression of glycosyltransferases impairing ganglioside synthesis, including ST3GAL5, ST8SIA3, B4GALNT1, and ST3GAL2 was observed. The same study showed decreased gangliosides’ concentrations in the diseased human caudate and the mouse striatum [364]. 

In contrast, patients with Alzheimer’s disease were found to display elevated levels of the gangliosides GM1, GM2, and GM3 in their cerebral cortices [360,365]. Amyloid-*β*, whose deposition defines the onset of Alzheimer’s, is bound to ganglioside species [366]. Speculations towards the neurotoxic effects of the insoluble GM1-bound amyloid-*β* were under study [367], suggesting that this particular binding aids in the formation of the insoluble *β*-pleated sheets [340]. Further studies on glycosyltransferases show that overexpression of B4GALNT1 causes increased ganglioside expression along with increased amyloid precursor protein (APP) that suppresses lysosomal degradation of *β*-secretase-1 (BACE1) and thus leads to amyloid-*β* pathology [368].

## 7. Potential Biomarkers in Disease Diagnosis (Clinical Application)

Finding potential biomarkers for any disease would be crucial in the early clinical diagnosis for this disease, then working toward treating or even eradicating it after discovering its whole mechanism. Clinical glycomics might play a major role in different medical areas and specializations since it can help unveil the glycosylation mechanisms [369]. This would be done through a set of several analytical methodologies that would determine and analyze the structure of any glycan such as gel electrophoresis, mass spectrometry, and from free glycans to intact glycoproteins [369], and more specialized ones as lectin capture methodology [370]. Moreover, discovering the combination of protein levels and their glycan isoforms would increase specificity for early diagnosis and therapy monitoring for several human disorders like cancer, inflammation, Alzheimer’s disease, and diabetes. This is because clinical validation is as important as acknowledging all the genetic and environmental factors which usually affect the protein-specific glycosylation abnormalities [371].

Wide applications were performed and studied on different potential biomarkers and medical fields. In the study of Chong et al., they tested a novel biomarker for inflammatory diseases, namely Leucine-rich alpha-2 glycoprotein (LRG). Although they studied other proteins and biomarkers that might affect inflammatory diseases, their results showed a relationship only between LRG and patients with either bacterial meningitis or aseptic meningitis [372]. 

Another study focused on finding a methodology that would allow them to get as much as they can from the biomarkers in the blood, which is considered a golden source for disease biomarkers. Thus, they performed prolonged ultracentrifugation coupled to electrostatic repulsion-hydrophilic interaction chromatography (PUC-ERLIC) to discover these biomarkers, and then they quantified them using mass spectrometry-based proteomic technique [373]. On the other hand, the accessibility of the skin was a target for other researchers to develop noninvasive tests of metabolic and disease activity for clinical use. Thus, they studied potential biomarkers for chronic inflammatory disorders as impaired human wound healing, and they were dermal extracellular matrix components such as collagens, proteoglycans, hyaluronan, and glycoproteins [374].

Each disease has specifically related biomarkers, and sometimes multiple biomarkers are expressed in several diseases, especially if the diseases are related to the same organ. For example, in some neurodegenerative diseases, some common biomarkers can be found, such as MOG, which is suggested as a potential biomarker for demyelinating diseases such as AD and MS [375]. Furthermore, other biomarkers such as apolipoprotein A-1, alpha-2-HS-glycoprotein, and afamin are only expressed in patients with AD [376]. Moreover, biomarkers such as glycoprotein non-metastatic melanoma B are considered a potential biomarker for Gaucher disease to monitor individual patients and even understand the disease mechanisms, which might give more insights into other related diseases through the clinical applications [377].

## 8. Conclusions

Intense multidisciplinary research and numerous studies have provided robust evidence of the potential role of glycomics to yield new classes of biomarkers for neurological and psychiatric disorders. Such tools can represent an expanded approach to an early and more accurate diagnose, improve patient characterization and classification, and elucidate novel pathogenetic and pathophysiological information to be translated into disease-modifying therapeutic strategies. Nonetheless, to make a wide impact on medical practice and support clinical decision making, well-validated platforms at a reasonable cost and with short turnaround times are needed, and clinical validity and utility of these new markers must be convincingly demonstrated in large rigorous and independent clinical studies.

## Figures and Tables

**Table 1:** The main types of PTMs: process, localization, targeted sites, and affected biological processes.

PTM	Process	Localization	Common Amino Acids/Sites Targeted	Cellular Processes Affected
Phosphorylation	The addition of one or more phosphate groups to the protein by kinases	Phosphorylation occurs in the nucleus or cytosol	In animal cells: serine, tyrosine, and threonine	DNA replication and transcription, cell movement, cell metabolism, apoptosis, environmental stress responses
Glycosylation	The addition of carbohydrate molecules to the polypeptide chain by glycoyltransferases	Glycosylation occurs in the endoplasmic reticulum (ER), Golgi apparatus or cytosol	Serine (Ser), threonine (Thr), asparagine (Asn), and tryptophan (Trp) residues	Cell adhesion, cell-cell, and cell-matrix interactions, receptor activation and signal transduction, protein secretion and trafficking
Acetylation	The addition of an acetyl group by acetyltransferase (KAT) and histone acetlytransferases (HAT)	Acetylation takes place mainly in the nucleus	Lysine (Lys) residues	Transcription regulation, protein-protein interaction, cell metabolism, nuclear transport
Sulfation	The addition of sulfate molecules by tyrosylprotein transferases (TPST)	Sulfation takes place in the trans-Golgi network	Tyrosine (Tyr) residues	Protein-protein interactions and leukocyte rolling
Hydroxylation	The addition of a hydroxy (OH) group to a protein amino acid by hydroxylases	Hydroxylation occurs in the cytosol	Lysine (Lys) and proline (Pro) residues	Transcription factor regulation
SUMOylation	The addition of SUMO protein via three enzymes (E1, E2 and E3)	SUMOylation occurs in the cytoplasm and nucleus	Lysine (Lys) residues	Transcription regulation and signal transduction
Ubiquitylation	The attachment of ubiquitin to a target protein by ubiquitin ligase and ubiquitin-conjugating enzyme	Ubiquitylation takes place in the cytosol	Lysine (Lys) residues	Protein degradation, transcription regulation, apoptosis and autophagy
Methylation	The transfer of a methyl group or more to amino acid side chains by methyltrasnferaes	Methylation usually occurs in the nucleus	Lysine (Lys) and arginine (Arg) residues	Histone modification, transcription regulation and epigenetic silencing

**Table 2:** A description of the different types of glycosylation.

Types of Glycosylation	
*N*-linked	Glycans bind to the amino group of asparagine in the ER
*O*-linked	Monosaccharides bind to the hydroxyl group of serine or threonine in the ER, Golgi, cytosol, and nucleus
*C*-linked	Mannose binds to the indole ring of tryptophan
Phospho-glycosylation	Glycan binds to serine via a phosphodiester bond

**Table 3:** Detailed descriptions of several neurodegenerative diseases, tackling the glycosylation changes occurring within each disease further exacerbating its consequences. These diseases include Alzheimer’s, Parkinson’s, Prion diseases, and many others.

Title	Neurodegenerative Disease	Glycosylation Aspect	Results	Analytical Methods	Ref.
Glyceraldehyde-3-phosphate dehydrogenase: Aggregation Mechanisms and Impact on Amyloid Neurodegenerative Diseases.	Amyloid neurodegenerative diseases	Glycolytic enzyme glyceraldehyde-3-phosphate dehydrogenase (GAPDH) has the ability to change the concentration of carbonyl compounds like glyceraldehyde-3-phosphate and methylglyoxal.	• Inhibition of glycolysis is due to the decreased activity of modified GAPDH. • Dysregulation of the cell metabo-lism is due to the compartmentalization of phosphorylated and glycated GAPDH and the replacement of active GAPDH in supramolecular complexes by its dena-tured form. • Blocking of the chaperone system by misfolded forms of modified GAPDH leads to the formation of amyloid struc-tures. • Denatured and modified GAPDH could mediate amyloid-like transition of susceptible proteins and peptides (amy-loid beta peptide, tau protein, al-pha-synuclein, prion, etc)	ELISA	[221]
Identification of an Intracellular Site of Prion Conversion.	Prion diseases	Cellular prion protein (PrP^C^) is a glycosyl-phosphatidyl-inositol (GPI) anchored glycoprotein that is able to misfold to a pathogenic isoform PrP^Sc^. PrP^Sc^ acts as the causative agent of prion diseases.	• Mis-folding PrP^C^ to PrP^Sc^ is a causa-tive agent of prion diseases. • Understanding where the conver-sion of PrP^C^ to PrP^Sc^ occurs in cells can help to clarify the cellular mechanism of the disease and it opens the door to new therapeutic strategies aimed at the con-version compartment. • It has been found that the prion conversion occurs in the endosomal recy-cling compartment (ERC), where it trans-its after being internalized from the cell surface.	Immunofluorescence	[222]
Alterations in Sulfated Chondroitin Glycosaminoglycans Following Controlled Cortical Impact Injury in Mice	Traumatic Brain Injury (TBI)	Many actions of chondroitin sulfate proteoglycans (CSPGs) in the central nervous system (CNS) are governed by the specific sulfation pattern on the glycosaminoglycan (GAG) chains attached to CSPG core proteins.	• It has been found that there are specific changes in the level and localization of CSPGs and CS-GAGs in response to TBI, with the predominant elevation in 4-sulfated GAG chain surrounding the injury core.	Immunoblotting Immunostaining	[223]
Increasing O-GlcNAc Slows Neurodegeneration and Stabilizes Tau against Aggregation.	Alzheimer’s disease (AD)	Oligomerization of tau is a key process contributing to the progressive death of neurons in AD. Tau is modified by *O*-linked N-acetylglucosamine (O-GlcNAc), and in some cases, O-GlcNAc can influence tau phosphorylation.	• It has been found that the treatment of hemizygous JNPL3 tau transgenic mice with and O-GlcNAcase inhibitor elevated tau O-GlcNAc, hindered the tau aggre-gates formation and diminished neuronal cell lost. • Based on the in vitro biochemical aggregation studies, it has been suggested that the O-GlcNAc may be to prevent pro-tein aggregation. • It is also suggested that O-GlcNacase can be considered as a po-tential therapeutic target that could hin-der progression of AD.	SDS-PAGE Western blot Fluorescence immunohistochemistry (IHC)	[224]
Mutation in B4GALNT1 (GM2 Synthase) Underlie a New Disorder of Ganglioside Biosynthesis.	Diseases of ganglioside biosynthesis	A mutation in the B4GALNT1 gene, encoding GM2 synthase, catalyzes the second step in complex ganglioside biosynthesis, as the cause of this neurodegenerative phenotype.	• Biochemical profiling of the glycosphingolipid biosynthesis confirmed that a lack of GM2 in affected subjects is associated with a predictable elevation in its precursor levels (GM3), which can significantly facilitate the diagnosis of this disease.	MALDI mass spectrometry Gas chromatography	[225]
Receptors for Advanced Glycosylation Endproducts in Human Brain: Role in Brain Homeostasis.	Alzheimer’s disease (AD) and other neurodegenerative diseases	Advanced glycation end products (AGEs) are the reactive of nonenzymatic glucose macromolecule condensation products, which play role in neuroinflammation.	• Non-enzymatic glycosylation is implicated in the theory of aging. This suggests the central role of advanced glycation end products in age-relation cognitive features.	Immunohistochemistry RT-PCR Western blot Ligand blot	[226]
Glycosylation Status of Nicastrin Influences Catalytic Activity and Substrate Preference of γ-Secretase.	Alzheimer’s disease	The assembly of nicastrin (NCT) and its maturation occurs through complex *N*-glycosylation including the terminal sialic acid residues on NCT glycan, affecting γ-Secretase complex.	• γ-secretase complex catalyzes the cleavage of amyloid precursor protein to generate amyloid-β pro-tein (Aβ), the main cause of Alz-heimer’s disease. • Complex glycosylation of NCT including terminal sialylation is critical for γ-secretase activity. • Immature NCT preferentially reduced Aβ generation in both cell-based and biochemical assays. • Thorough glycosylation of NCT is critical for enzymatic activi-ty and substrate preference of γ-secretase.	Gel electrophoresis Western blot	[227]
Glycosylation Significantly Inhibits the Aggregation of Human Prion Protein and Decreases Its Cytotoxicity.	Prion diseases	Wild-type PrP and its monoglycosylated mutants N181D, N197D, and T199N/N181D/N197D are primarily attached to the plasma membrane via a glycosylphosphatidylinositol (GPI) anchor. This glycosylation occurs at 2 sites being Asn-181 and Asn-197 at the C-terminal through sialylation.	• Glycosylation deficiency enhances human prion protein (PrP) cytotoxicity induced by MG132 or the toxic prion peptide PrP 106–126. • Glycosylation acts as a necessary cofactor in determining PrP localization on the plasma membrane and that it significantly inhibits the aggregation of human PrP and decreases its cytotoxicity.	Western blotting Flow cytometry Circular dichroism spectroscopy Laser scanning confocal analysis	[228]
Disruption of Golgi Morphology and Altered Protein Glycosylation in PLA2G6-associated Neurodegeneration.	PLA2G6-associated neurodegeneration (PLAN)	*N*-linked and *O*-linked glycosylation in cerebrospinal fluid, plasma, urine, and cultured skin fibroblasts were assessed, along with sialylation and Golgi morphology in cultured fibroblasts.	• Golgi morphology, *O*-linked glycosylation and sialylation may play a role in the pathogenesis of PLAN and perhaps other neurodegenerative disorders. • Alteration in Golgi morphology and abnormalities of protein *O*-linked glycosylation and sialylation in cultured fibroblasts were rescued by lentiviral overexpression of wild type PLA2G6.	HPLC MALDI-TOF/MS Immunofluorescence Lentiviral vector	[38]
Sialylation Enhances the Secretion of Neurotoxic Amyloid-β Peptides.	Alzheimer’s disease	Overexpression of the *β*-galactoside *α*2,6-sialyltransferase (ST6Gal-I) in Neuro2a cells enhances *α*2,6-sialylation of endogenous APP and increases the extracellular levels of its metabolites.	• In the mouse model, the amount of *α*2,6-sialylated amyloid precursor protein (APP) appeared to be correlated with the soluble APP*β* (sAPP*β*) level. • It is suggested that the sialylation of APP promotes its metabolic turnover and could affect the AD pathology.	Western blot	[229]
Loss of O-GlcNAc Glycosylation in Forebrain Excitatory Neurons Induces Neurodegeneration.	Alzheimer’s disease	Problems in O-GlcNAc glycosylation (or O-GlcNAcylation) of proteins like α-synuclein, amyloid precursor protein (APP), and tau in forebrain excitatory neurons may induce neurodegeneration diseases.	• O-GlcNAc modification plays a central role in regulating both APP and tau and that dysfunctional O-GlcNAc signaling may contribute to improper APP processing and tau pathology. • O-GlcNAcylation levels can enhance nonamyloidogenic processing of APP by raising α-secretase activity and lowering γ-secretase activity. • O-GlcNAcylation regulates pathways critical for the maintenance of neuronal health and suggest that dysfunctional O-GlcNAc signaling may be an important contributor to neurodegenerative diseases.	Immunohistochemistry (IHC) ELISA Gene expression microarray qRT-PCR	[230]
V232M Substitution Restricts a Distinct O-glycosylation of PLD3 and its Neuroprotective Function.	Alzheimer’s disease	O-glycosylation at a specific site pT271 in phospholipase D3 (PLD3) is crucial for the wild-type’s normal trafficking and cellular localization. The Val232Met variant substitution impairs this O-glycosylation.	• Mutation of Val232Met variant of phospholipase D3 (PLD3) may affect AD pathogenesis by impairing this O-glycosylation, subsequently leading to enlarged lysosomes and possible aberrant protein recycling. • PLD3VM had a less neuroprotective function, while PLD3WT expression enhanced lysosomal functions, V232M weakened PLD3’s trafficking to the lysosomes.	Quantitative PCR Flow cytometry Cell immunocytochemistry	[231]
Glycation Potentiates α-Synuclein-associated Neurodegeneration in Synucleinopathies.	Parkinson’s disease and other neurodegenerative diseases	Glycation of the N-terminal region of α-synuclein by glucose is considered an age-associated post-translational modification. This PTM enhances α-synuclein toxicity in vitro and in vivo, in Drosophila and in mice.	• A hallmark present in Parkinson’s disease as well as other neurodegenerative diseases is α-synuclein misfolding and aggregation. • Glycation leads to reducing membrane binding of α-synuclein, impairing the clearance, and supporting the accumulation of toxic oligomers, that in turn impair neuronal synaptic transmission. • The use of glycation inhibitors allowed normal clearance of α-synuclein to be re-established, where the aggregations were reduced, alleviating the motor phenotypes in Drosophila.	Flow cytometry Immunoblotting Reverse phase HPLC Mass spectrometry Size exclusion chromatography Nuclear magnetic resonance spectrometry	[232]
The Prion Protein Requires Cholesterol for Cell Surface Localization.	Prion diseases and neurodegenerative disorders like Alzheimer’s disease	PrP^C^ is a cell surface glycoprotein linked to the outer leaflet of the plasma membrane by a glycosyl-phosphatidyl-inositol (GPI) anchor. Prion conversion from PrP^C^ to PrP^Sc^ occurs in the presence of cholesterol allowing prion propagation.	• Levels of cholesterol in the brains of affected individuals increase during the clinical course of both prion diseases and Alzheimer’s disease. • Imbalance in cholesterol homeostasis may lead to synaptic dysfunction and neurodegeneration in prion diseases and AD.	Immunoblot	[233]
Characterization of the Glycosylation Profiles of Alzheimer’s *β*-Secretase Protein Asp-2 Expressed in a Variety of Cell Lines.	Alzheimer’s disease	Asp-2 is a transmembrane aspartic protease expressed in the brain, shown to have *β*-secretase activity. Mature Asp-2 has four *N*-glycosylation sites.	• Carbohydrate structure characterization of Asp-2 expressed in Chinese hamster ovary, CV-1 origin of SV40, and baculovirus-infected SF9 cells were reported. • It has been reported that biantennary and triantennary oligosaccharides of the complex type were released from glycoproteins expressed in the mammalian cells, while high mannose glycan types were identified from glycoprotein synthesized in the baculovirus-infected cells. • Protease activity of Asp-2 is depended on its glycosylation.	Gel electrophoresis HILIC MALDI-TOF-MS	[234]
Altered Protein Glycosylation Predicts Alzheimer’s Disease and Modulates its Pathology in Disease Model Drosophila.	Alzheimer’s disease	The process of capping N- and *O*-linked glycans by a terminal sialic acid (sialylation) was reported to be altered in AD. Inhibiting the MGEA5 gene, encoding the enzyme that dynamically removes O-GlcNAc from proteins, OGA, reduces the extent of O-GlcNAc removal from tau.	• Many glycosylation-related genes are differentially expressed in brains of AD patients compared with healthy controls. • The result from the in vivo study in AD model indicates that certain alterations in expression levels of glycosylation-related genes are casually related to disease severity, whereas others are circumstantial.	Western blot	[235]
A Comprehensive Glycome Profiling of Huntington’s Disease Transgenic Mice.	Huntington’s disease (HD)	Total glycomics, namely, N-glycomics, O-glycomics and glycosphingolipidomics of HD transgenic mice can be a hallmark for the central nervous system disorders to discover disease biomarkers.	• Core-fucosylated and bisecting-GlcNAc types of N-glycans were found to be over expressed in the brain tissue HD mice. • Core-fucosylated and sialic acid for biantennary type glycans were found to be elevated in the sera of HD transgenic mice compared to the control mice. • Core 3 type O-glycans were increase in male and decrease in both striatum and cortexes of female HD transgenic mice. • Serum levels of core 1 type O-glycans decreased and core 2 type o-glycans were undetected for HD transgenic mice. • In glycosphingolipids, GD1 increased in brain tissue, and GM2-NeuGc decreased in serum.	Glycoblotting MALDI-TOF/MS	[236]
Interplay between Protein Glycosylation Pathways in Alzheimer’s Disease.	Alzheimer’s disease	Serum samples of 10 AD patients, MCI patients, and controls were studied.	• Differences in levels of glycan involved in both protein O-GlcNAcylation and N-/O-glycosylation between patients and healthy individuals can be seen, revealing brain region–specific glycosylation-related pathology in patients. • Robust decrease in protein O-GlcNAcylation and elevation in PAS staining of the soluble fraction of frontal cortex tissue of AD patients can be observed when compared to that in healthy controls. • Glycosylation alterations identified by PAS staining in the soluble membrane fractions of AD patients could be partially attributed to alterations in glycosylation of molecules other than glycoproteins, such as glycolipids. • The alterations in the AD glycome in the serum could potentially lead to novel glyco-based biomarkers for AD progression.	SDS-polyacrylamide gel electrophoresis, Western blot ELISA Lectin chip microarray	[237]

**Table 4:** An overview of the alterations in the normal glycosylation patterns occurring in Schizophrenia, a neuropsychiatric disorder, along with the subsequent consequences on other protein expression levels.

Title	Neurodegenerative Disease	Glycosylation Aspect	Results	Analytical Methods	Ref.
Abnormal *N*-acetylglucosaminyltransferase Expression in Prefrontal Cortex in Schizophrenia.	Schizophrenia	*N*-linked and *O*-linked glycosylation in cerebrospinal fluid (CSF) and plasma along with glycosyltransferase transcripts in frontal cortex were studied. Comparison of protein expression of nine *N*-acetylglucosaminyltransferases (GlcNAcTs) glycosylating enzymes in postmortem tissue from the dorsolateral prefrontal cortex of 12 elderly patients with schizophrenia and 12 healthy controls was done.	• There was a decrease in protein expression of UDP-GlcNAc: BetaGal Beta-1, 3 GlcNAcT 8 (B3GNT8) and mannosyl (alpha-1, 3-)-glycoprotein beta-1, 4 GlcNAcT (MGAT4A) expression in patients with schizophrenia compared to controls, providing evidence for dysregulated glycosylation in schizophrenia.	Western blot	[270]
*N*-linked Glycosylation of Cortical *N*-methyl-D-aspartate and Kainate Receptor Subunits in Schizophrenia.	Schizophrenia	*N*-glycosylation of ionotropic glutamate receptors (iGluRs) and *N*-glycosylation of *N*-methyl-*D*-aspartate (NMDA) and kainate (KA) receptor subunits in the dorsolateral prefrontal cortex was studied. Comparison of NMDA and kainate receptor subunits *N*-glycosylation in postmortem tissue from the dorsolateral prefrontal cortex of 35 patients with schizophrenia and 31 healthy controls was performed.	• The levels of NMDA and kainite receptor subunits were unchanged between patients with schizophrenia and healthy controls. • NR1, NR2A, and NR2B NMDA receptor subunits, and GluR6 and KA2 kainate receptor subunits were *N*-glycosylated. • GluR6 was significantly more sensitive to endoglycosidase H in patients with schizophrenia, reflecting a large molecular mass of *N*-linked high mannose and/or hybrid sugars on the GluR6 protein subunit in patients with schizophrenia	SDS-polyacrylamide gel electrophoresis	[271]
Abnormal *N*-linked Glycosylation of Cortical AMPA Receptor Subunits in Schizophrenia.	Schizophrenia	*N*-linked glycosylation occurs in the ER and the Golgi apparatus before the assembled receptors are transported to the plasma membrane. Comparison of AMPA receptor subunit *N*-glycosylation in postmortem tissue from the dorsolateral prefrontal cortex of 35 schizophrenia patients and 31 healthy controls was done.	• The absolute level of AMPA receptors may not be critical, but rather changes in trafficking and activity of these receptors may contribute to schizophrenia.	Western blot Lectin-binding assays Immunoisolation	[272]
*N*-Glycosylation of GABAA Receptor Subunits is Altered in Schizophrenia.	Schizophrenia	*N*-glycosylation of molecules associated with glutamatergic neurotransmission were checked. Comparison of *γ*-aminobutyric type A receptor (GABA_A_R) subunit *N*-glycosylation in postmortem tissue from the superior temporal gyrus of 14 adult patients with schizophrenia and 14 healthy controls was performed.	• There was evidence for *N*-glycosylation of the *α*1, *β*1, and *β*2 GABA_A_R subunits in patients with schizophrenia, with characteristic glycan attachment on the *α*1, *α*4, and *β*1–3 GABA_A_R subunits. • Although the *N*-glycosylation of *α*1, *β*1, and *β*2 were all changed in patients with schizophrenia, the concentrations of GABA_A_R subunits themselves were unchanged.	Western blot Lectin Affinity Isolation	[273]
Antipsychotic Treatment of Acute Paranoid Schizophrenia Patients with Olanzapine Results in Altered Glycosylation of Serum Glycoproteins.	Schizophrenia	Disialylated bi- and triantennary glycans were checked. Identification of the glycosylation profile of serum proteins in 23 antipsychotic-naïve adult patients diagnosed with acute paranoid schizophrenia before and after 6 weeks of treatment with Olanzapine was performed.	• It has been shown that olanzapine treatment of schizophrenia patients resulted in changes in the glycosylation machinery associated with the biosynthesis of abundant serum proteins. • Olanzapine appeared to affect the extent of digalactosylation and disialylation of serum proteins. • As glycosylation impacts on many important cellular processes, olanzaoine-induced glycosylation changes may induce a number of downstream effects	HILIC fluorescence-based glycoanalytical technology Two-dimensional gel electrophoresis SDS-PAGE gel electrophoresis MALDI-TOF Mass Spectrometry ELISA	[274]
Identification of *N*-glycosylation Changes in the CSF and Serum in Patients with Schizophrenia.	Schizophrenia	*N*-glycans and sialylated glycans in the cerebrospinal fluid (CSF) appear altered in schizophrenia patients. Comparison of serum and CSF glycans of adult patients with first onset unmedicated schizophrenia (19 for serum and 14 for CSF) and healthy controls (19 for serum and 18 for CSF) was done.	• Changes in protein glycosylation are associated with disease physiopathology, with some of the alterations being gender specific, and can be hold potential as diagnostic tools for schizophrenia.	NP-HPLC	[275]
Abnormal Glycosylation of EAAT1 and EAAT2 in Prefrontal Cortex of Elderly Patients with Schizophrenia.	Schizophrenia	*N*-glycosylation can regulate excitatory amino acid transporters (EAATs). Comparison of the glycosylation pattern of EAATs in postmortem tissue from the dorsolateral prefrontal and anterior cingulate cortices of 35 adult patients with schizophrenia and 33 healthy controls was performed.	• There is significantly less glycosylation of both EAAT1 and EAAT2 (glial transporters) in neuronal postmortem tissues of patients with schizophrenia. • There was no evidence for *N*-linked glycosylation of EAAT3 (neuronal transporter) in postmortem tissues of either patients with schizophrenia or healthy controls. • Deficits in glycosylation that are glia-specific may have a role in the pathophysiology of schizophrenia.	Gel Electrophoresis Western blot	[276]
Evidence for Disruption of Sphingolipid Metabolism in Schizophrenia.	Schizophrenia	This study compares the expression of genes encoding proteins related to glycobiology in the prefrontal cortex, related to *N*- and *O*-linked glycan biosynthesis of 30 adult patients with schizophrenia and 30 healthy controls.	• There was a statistically significant decrease in the expression of seven genes encoding for glycan transferases in the *N*- and *O*-linked glycan biosynthetic pathways and glycosphingolipid metabolism in patients with short-term illness, and one gene in those with chronic illness.	Spectrophotometer Microarray Analysis PCR	[277]
Serum Glycoconjugates in Children with Schizophrenia and Conduct and Adjustment Disorders.	Schizophrenia	Glycoproteins and glycosaminoglycans are altered in the sera of children. Comparison of serum glycoproteins in 8 children with schizophrenia, 11 with conduct disorder, 6 with adjustment disorder and 20 13–17 years of age healthy controls was conducted.	• The serum glycosaminoglycans were significantly elevated only in children with schizophrenia (versus normal range in the three other groups). • The protein-bound carbohydrates were all significantly elevated in children with schizophrenia (versus only arabinose and galactosamine in children with conduct disorder, and only galactosamine in children with adjustment disorder).	Chemical ionization-mass spectrometry	[278]
Serum Glycoproteins in Schizophrenia.	Schizophrenia	Serum glycoproteins containing glucose and *L*-arabinose, in addition to mannose, galactose, fucose, sialic acid, and a trace of xylose are examined. Comparison of serum glycoproteins and their carbohydrate component in 30 adult patients with schizophrenia and 20 healthy controls was performed.	• The mean concentration of each of the protein-bound carbohydrate components was significantly elevated in patients with schizophrenia • The electrophoretic patterns for serum glycoprotein showed increases in alpha-2 and beta globulins in patients with schizophrenia. • The contents of glucose and arabinose were higher in serum glycoproteins from patients with schizophrenia.	GLC-electron-impact mass spectrometry	[279]
